# Supplementary material for: RNACOREX - RNA coregulatory network explorer and classifier
Source: PLoS Comput Biol. 2025 Nov 3;21(11):e1013660. doi: 10.1371/journal.pcbi.1013660 (PMC12594346; doi:10.1371/journal.pcbi.1013660)
Supplement: S1 Text — Table A. Engine nomenclatures.​ Table B. Engine composition.​ Fig A. Engine overlap.​ (PDF) [file pcbi.1013660.s001.pdf]

Engines

The RNACOREX tool is guided by four key resources (also called engines in the package), two prediction databases, TargetScan v8.0 [1] and DIANA-microT 2023 [2], and two experimentally validated interaction databases, miRTarBase v10.0 [3] and TarBase v9.0 [4]. These databases collectively enable the identification of the potential interaction set of miRNA–mRNA interactions (**S**). RNACOREX filters out interactions that are not supported by any of these databases, under the assumption that their absence indicates insufficient scientific support and may result in false positives.

TargetScan v8.0 and DIANA-microT-CDS are widely used computational tools for predicting microRNA (miRNA) targets, each employing distinct methodologies. TargetScan identifies canonical seed matches (8mer, 7mer, 6mer) primarily within the 3' untranslated regions (3' UTRs) of mRNAs and calculates a context++ score. This score integrates multiple binding site features, such as site type, 3' supplementary pairing, local AU content, site accessibility, position within the UTR, and evolutionary conservation, to estimate the likelihood and efficacy of miRNA-mediated repression. In contrast, DIANA-microT-CDS expands the search to include both 3' UTR and coding sequence (CDS) regions, increasing the potential target space. Rather than relying on a single contextual score, DIANA-microT generates a confidence score by combining diverse factors including sequence complementarity, binding free energy, evolutionary conservation, as well as additional functional evidence such as co-expression patterns and pathway involvement. This integration is achieved through a machine learning framework that weighs these features to provide a robust prediction score. Consequently, DIANA-microT-CDS tends to identify a broader spectrum of potential targets, encompassing both well-established and less obvious functional interactions.

Regarding experimentally validated interactions, miRTarBase manually curates miRNA-target interactions from the scientific literature, classifying them according to the type of experimental validation and distinguishing between strong and weak evidence. In contrast, TarBase also compiles validated interactions but organizes experimental evidence into low-throughput and high-throughput categories. Both databases incorporate interactions identified through high-throughput techniques; however, TarBase systematically and broadly includes these data, often integrating results from large-scale experiments such as CLIP-seq and RNA-seq. Conversely, miRTarBase includes high-throughput data only when there is clear experimental support linking a specific miRNA-mRNA interaction reported in published studies. Consequently, TarBase offers more inclusive and comprehensive coverage of potential interactions, including many derived from large-scale screenings, while miRTarBase provides a more stringent and detailed curation focused on functionally validated interactions. This difference reflects the complementary nature of these resources: TarBase is valuable for exploring broad interaction landscapes, whereas miRTarBase is preferred when high-confidence, experimentally confirmed targets are required.

- **Engine curation**

Since each database has its own methodology for data collection, processing, and identifier assignment, the process of integrating databases is not straightforward.

Across the four engines, three different nomenclatures are used to identify miRNAs: miRBase name (*hsa-mir-XXX*), miRBase ID (*MIMATXXX*) and miRTarBase ID (*MIRTXXX*, specific to miRTarBase). Because all engines reference the miRBase name, this identifier was chosen as the standard for unifying miRNA nomenclature. The miRBase name may refer either to a precursor (e.g., *hsa-mir-42*) or to a mature miRNA (e.g., *hsa-miR-42-3p*). Interactions are preserved as reported in the original engines, with miRNAs identified either by precursor or mature identifiers according to the corresponding source database.

In contrast, mRNAs are represented using Ensembl and/or HUGO Symbol nomenclatures. Within Ensembl, identifiers may appear at either the gene level (e.g., *ENSGXXXXXX*) or the transcript level (e.g., *ENSTXXXXXX*) and may include version numbers (e.g., *ENSGXXXXXX.XX*) or not (e.g., *ENSGXXXXXX*). For normalization, the gene-level identifiers are adopted, since transcript IDs can be reliably mapped to gene IDs (with some loss of specificity), whereas the reverse is not possible. Moreover, stable Ensembl identifiers (ID without version numbers) are used, as different versions correspond to the same biological gene and can therefore be generalized across interactions. In practice, this means that in TargetScan, only the gene-associated ID is retained and the version number is

discarded (*ENSGXXXXXX*). In TarBase, the gene-level identifier (without version) is also used, while transcript-level identifiers are excluded. In miRTarBase, identifiers are first provided in HUGO Symbol nomenclature and then mapped to their corresponding Ensembl gene IDs using the GENCODE v47 annotation database. Table A summarizes the identifier formats and characteristics as they appear in the different databases.

Table A. Engine nomenclatures.

	Element	TargetScan	DIANA	miRTarBase	TarBase
miRBase name	microRNA	Yes	Yes	Yes	Yes
miRBase ID	microRNA	No	No	No	Yes
miRTarBase ID	microRNA	No	No	Yes	No
Ensembl	mRNA	Yes	Yes	No	Yes
HUGO	mRNA	No	No	Yes	Yes
Transcript	mRNA	Yes	No	No	Yes
Version	mRNA	Yes	No	No	No

Table notes. The table shows which nomenclatures are present in each engine. The **Element** column refers to which RNA molecule the nomenclature is referred. **miRBase name:** If *'hsa-mir-XXX'* nomenclature is present for miRNAs or not. **miRBase ID:** If *'MIMATXXX'* nomenclature is present for miRNAs or not. **miRTarBase ID:** If miRTarBase identifier (*'MIRTXXX'*) is present for miRNAs or not. **Ensembl:** Shows if the mRNA is represented with Ensembl nomenclature (*'ENSG/ENST'*). **HUGO:** Represents if the mRNA is identified with the HUGO Symbol. **Transcript:** Represents if the mRNA is also represented using the Ensembl nomenclature at transcript level. **Version:** Shows if the version number is represented within the Ensembl nomenclature for the mRNAs.

After selecting only the 'Homo Sapiens' related interactions and developing the data curation as defined above, Table B shows the characteristics of the four engines.

Table B. Engine composition.

	TargetScan	DIANA	miRTarBase	TarBase
Raw interactions	14.3M	25.2M	4M	4.72M
Unique interactions	10M	25.2M	1.7M	1.3M
Specific interactions	1.45M (14.6%)	16.1M (63.89%)	400k (23.58%)	371k (28.06%)
Mean presence	2	1.42	2.03	2.48

Table notes. Raw Interactions. Number of interactions in each database after selecting *'homo-sapiens'* related entries only. **Unique interactions.** Number of interactions in each engine after removing duplicated entries. **Specific interactions.** Number of interactions that only have presence in the database. **Mean presence.** Average number of engines in which interactions of the database appear.

In addition to this information, it is worth noting that 18 million interactions are specific to a single database, 7.7 million are shared by two databases, 1.4 million by three, and approximately 50,000 are present across all four databases. Several aspects of these data deserve comment. Regarding validated and predicted interactions, TarBase and DIANA employ more inclusive methodologies compared to miRTarBase and TargetScan. Consequently, a higher number of raw interactions can be expected in the former two resources. For example, DIANA reports a substantially larger number of unique interactions than TargetScan (25.2M vs. 10M). In contrast, the situation is reversed for TarBase and miRTarBase: miRTarBase includes more interactions than TarBase (1.7M vs. 1.3M). This discrepancy is likely due to differences in release dates, as miRTarBase v10 was recently published, whereas TarBase v9 is comparatively older. The mean presence of the interactions show how the validated interactions are more widely represented in the engines than the predicted interactions. In Fig A, the overlapping between databases is represented.

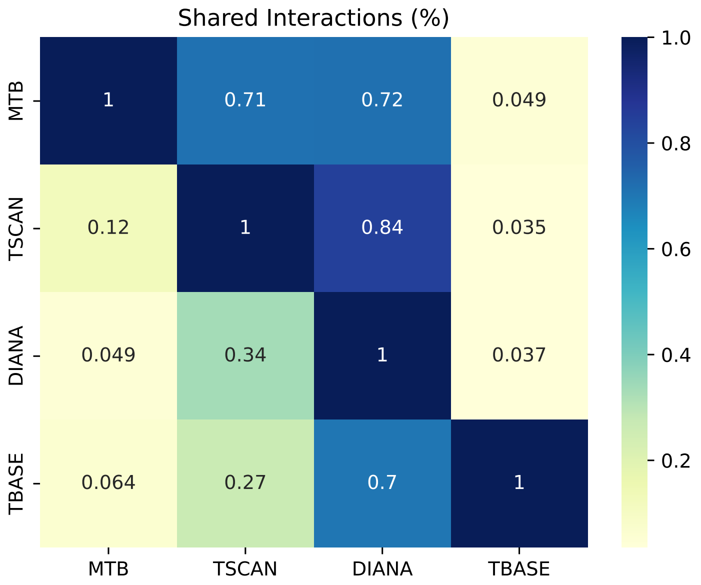

Fig A: Engine overlap. The Figure shows the proportion of shared interactions between engines. **MTB**: miRTarBase, **TSCAN**: TargetScan, **DIANA**: DIANA microT CDS, **TBASE**: TarBase.

It is logical to expect TarBase and miRTarBase, as sources of experimentally validated interactions, to share a subset of interactions. Anyway, the actual overlap is limited to approximately a 5%. This discrepancy arises from the different curation strategies applied by each database. miRTarBase extracts validated interactions directly from scientific publications, recording them as they appear in the original articles. Using this approach, older references often report miRNAs in their precursor form (without specifying the 3p/5p strand). In contrast, TarBase performs a posteriori curation, incorporating the mature miRNA form whenever possible. Since interactions are preserved in RNACOREX exactly as reported in the original engines, these methodological differences lead to substantial discrepancies between the validated sources, with TarBase containing a higher proportion of mature miRNAs than miRTarBase. Anyway, DIANA covers approximately 70% of the interactions from both validated sources, mitigating potential information loss during RNACOREX's filtering process. This small overlapping between experimentally validated sources is not new and have been commented in recent studies [5].

References

[1] Agarwal V, Bell GW, Nam J-W, Bartel DP. Predicting effective microRNA target sites in mammalian mRNAs. *Computational and Systems Biology* 2015.

[2] Tastsoglou, S, et al. DIANA-microT 2023: including predicted targets of virally encoded miRNAs. *Nucleic Acids Research.* 2023; 51: W148–W153.

[3] Cui S, et al. miRTarBase 2025: updates to the collection of experimentally validated microRNA-target interactions. *Nucleic Acids Research.* 2025 Jan 6; 53(D1): D147-D156.

[4] Skoufos G, Kakoulidis P, Tastsoglou S. TarBase-v9.0 extends experimentally supported miRNA–gene interactions to cell-types and virally encoded miRNAs. *Nucleic Acid Research.* 2024, Vol. 52, pp. 304-310.

[5] Kariuki D., Asam K., Aouizerat B. E., Lewis K. A., Florez J. C., Flowers E. Review of databases for experimentally validated human microRNA–mRNA interactions. *Database.* 2023 Apr.
