# Supplementary material for: RNACOREX - RNA coregulatory network explorer and classifier
Source: PLoS Comput Biol. 2025 Nov 3;21(11):e1013660. doi: 10.1371/journal.pcbi.1013660 (PMC12594346; doi:10.1371/journal.pcbi.1013660)
Supplement: S2 Text — Fig A. CMI Estimations.​ Fig B. CMI Standard Deviation.​ Fig C. CMI execution time.​ Fig D. Tie-breaking procedures.​ (PDF) [file pcbi.1013660.s002.pdf]

Theoretical Background

Structural information score

The potential interaction set, containing all considered interactions after the filtering step, is defined as $\mathbf{S}$. For each potential interaction, its *structural information score* will depend on two elements: the number of engines in which it is present and the *consistency index* between the pairs of engines in which it appears. The *consistency index*, as described by Armañanzas et. al [1], is a measure of similarity between groups, adapted from Kuncheva's original measure [2]. For each engine, the content of each database can be reduced to a list of micro-gene pairs $\mathbf{S_i} \subseteq \mathbf{S}$ with their predicted or validated interactions. For each pair of engines (S_k, S_l), the consistency index $I(\mathbf{S_k}, \mathbf{S_l})$ can be calculated.

$$I(\mathbf{S_k}, \mathbf{S_l}) = \frac{rn - k_M^2}{k_M(n - k_M)}. \quad (1)$$

With $n = |\mathbf{S}|$ the number of interactions in the potential interaction set, $k_k = |\mathbf{S_k}|$ and $k_l = |\mathbf{S_l}|$ the cardinalities of the two subsets and $r = |\mathbf{S_k} \cap \mathbf{S_l}|$ the cardinality of the intersection between the subsets. $k_M = max\{k_k, k_l\}$ corresponds to the number of elements in the largest subset. RNACOREX calculates the consistency index for each pair of engines. All consistency indices are normalized so that they all add 1. This index aims to increase the relevance of an interaction if the databases in which the interaction is present are consistent with each other.

Once the consistency index for each pair of engines is calculated, it is used for computing the *structural information score* of each interaction. This score is calculated by multiplying the *consistency index* of each pair of engines with a binary variable w_{ij}^{kl} representing the presence of a specific (i, j) interaction in a pair of engines.$(\mathbf{S_k}, \mathbf{S_l})$. This binary variable will be assigned to $w_{ij}^{kl} = 0$ if the interaction does not appear in both of the engines and $w_{ij}^{kl} = 1$ if it is present in both of them. For a (i, j) interaction, the final value of its *structural information score* (SI_{ij}) will be simply the sum of the normalized consistency indices between the pair of engines in which it is present.

$$SI_{ij} = \sum_{k=1}^{4} \sum_{l=1}^{4} I_{norm}(S_k, S_l) * w_{ij}^{kl}. \quad (2)$$

The values of the *structural information score* are also represented in a N dimensional matrix. 0 values will represent non-relevant interactions, while larger than zero values will correspond to relevant interactions. This matrix will be named as the *structural information matrix.*

Functional information score

The *functional information score* represents the empirical association between miRNA and mRNA expression conditioned to the class variable (supervised or target variable). This association is calculated using the miRNA and mRNA expression data from the input database defined by the user. In RNACOREX, this association is computed through the conditional mutual information encoded in each miRNA-mRNA-class triplet.

- **Conditional Mutual Information**

Mutual information is a non-negative symmetric measure of dependence between two random variables. Specifically, mutual information $CMI(X, Y)$ encodes the amount of information that one random variable X contains about another random variable Y [3]. When X and Y are continuous variables and C is discrete, the conditional mutual information $CMI(X, Y|C)$ can be computed as the information that X and Y jointly contain about the values in C.

$$CMI(X, Y|C) = \sum_{c \in \Omega_c} \int_Y \int_X p(c) f_{XY}(x, y|c) \log \frac{f_{XY}(x, y|c)}{f_X(x|c) f_Y(y|c)} dxdy. \quad (3)$$

In our case, X and Y are related to the miRNA and mRNA expression values, whereas C contains the phenotype or class. Higher values of $CMI(X, Y|C = c)$ show a stronger relation between X and

Y conditioned to c, suggesting a potentially significant interaction between miRNA X and mRNA Y in samples characterized by the phenotype c. Unfortunately, equation (3) has no closed form for continuous variables and the density functions have to be estimated using kernel methods.

- **Kernel Density Estimation**

Kernel density estimation methods (KDE) are used for estimating underlying probability density functions of a dataset in a non parametric way [4]. KDEs learn the shape of the distribution from the data automatically without any assumptions about the underlying distribution [5]. For unidimensional distributions, given a dataset $D = \{x_i, x_{i+1}, ..., x_n\}$ the kernel density estimator is defined as follows.

$$\hat{f}_h(x) = \frac{1}{nh} \sum_{i=1}^{n} K(\frac{x - x_i}{h}), \tag{4}$$

where $K(\cdot)$ is the smooth function (kernel function) and $h > 0$ is the bandwidth parameter that controls the amount of smoothing [5]. RNACOREX uses a Gaussian Kernel by default, with the bandwidth parameter set using the Scott's approximation of the normal reference rule [6].

$$h = \sigma_i^2 n^{\frac{-1}{d+4}}. \tag{5}$$

This equation can be generalized to the d-dimensional case where $\mathbf{H}$ maps a d dimensional square bandwidth matrix.

$$\hat{f}_{\mathbf{H}}(\mathbf{x}) = \frac{1}{n} \sum_{i=1}^{n} K_{\mathbf{H}}(x - x_i). \tag{6}$$

When estimating a distribution with kernels, it is well known that the election of the kernel function is not specially relevant [7], however the bandwidth method is [8]. RNACOREX offers the possibility of selecting alternative bandwidth methods, such as Silverman [9], or any other personalized function. RNACOREX implements the `gaussian_kde` function from `SCIPY` [10], and is compatible with all other bandwidth methods available in `SCIPY`.

- **Value approximation**

The conditional mutual information is computed using a numerical integration method, by approximating the integral of the KDE estimated density functions through the trapezoidal rule [11]. The trapezoidal rule works approximating the region under the curve of $f(x)$ in a $[a, b]$ interval by computing the area of its related trapezoid as follows.

$$\int_a^b f(x)\ dx \approx (b - a) \cdot \frac{1}{2}(f(a) + f(b)). \tag{7}$$

The area can be better approximated by partitioning the integration interval in several sub-intervals. An $[a, b]$ interval can be divided in k intervals as $a = x_0 < x_1 < ... < x_{n-1} < x_n = b$. Being Δx_k the length of the k-th sub-interval, if the length of the intervals is uniform, then the area under the curve can be approximated by adding the area of the trapezoid under every interval.

$$\int_a^b f(x)\ dx \approx \sum_{k=1}^{N} \frac{f(x_{k-1}) + f(x_k)}{2} \Delta x_k \tag{8}$$

The number of intervals used in the estimation process, and therefore the number of trapezoids, will directly determine the precision and complexity of the approximation, increasing precision at the cost of simultaneously increasing complexity as the number of trapezoids increases. For user convenience, RNACOREX uses by default 20 trapezoids. In order to justify the default choice, an in-depth analysis of both execution times and estimation accuracy of CMI is developed. The results can be consulted in 'Precision Setting'. The value of the estimated CMI for a (i, j) interaction will be defined as the *functional information score* FI_{ij} of the interaction.

The conditional mutual information will be calculated only for those miRNA-mRNA interactions with a non-zero *structural information score*. With the conditional mutual information calculated

for all miRNA-mRNA pairs, the functional information matrix is constructed, including the mutual information of each miRNA-mRNA interaction. These values will be the *functional information scores* of the interactions. In order to reduce running time, the *functional information score* will be calculated only for those interactions with a non-zero *structural information score*, setting the rest to 0.

- **Precision setting**

An in-depth analysis of both execution times and estimation accuracy of conditional mutual information (CMI) have been developed in order to justify the default choice of the precision values. Specifically, we performed computations on 50 RNA molecule pairs using precision values ranging from 1 to 50 trapezoids. In Fig A, we present the estimated CMI values for 10 of these pairs.

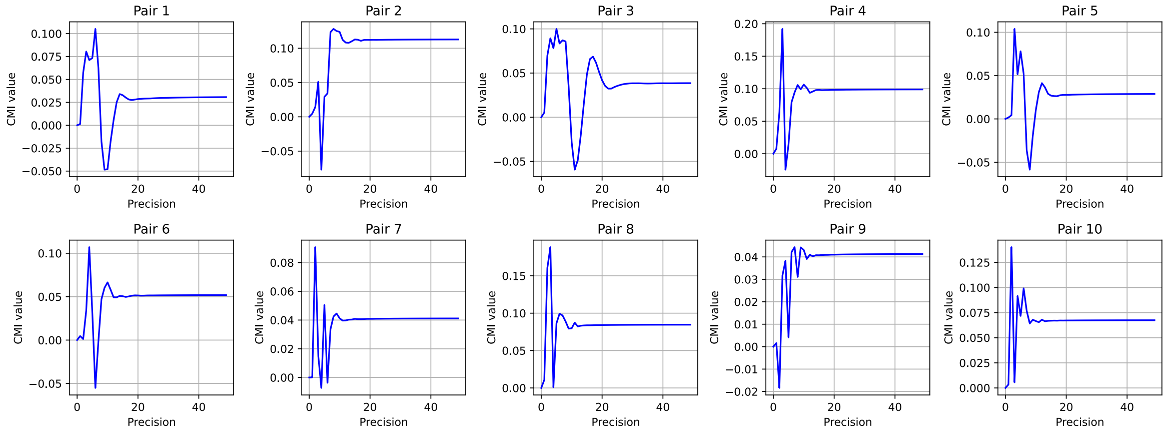

Fig A: CMI Estimations. Values of the estimated CMI for 10 randomly selected interactions with precisions (number of trapezoids) ranging from 0 to 50.

The conditional mutual information of each pair of elements is estimated using varying levels of precision (number of trapezoids) ranging from 1 to 50. These values eventually stabilize, converging to the true conditional mutual information, but the point of convergence differs for each pair of elements. To identify the point at which convergence can reasonably be assumed, we apply a rolling window of size three over the normalized CMI estimates and compute the standard deviation within each window. The intuition is that, as the estimates approach their true value, the variation within the rolling window will decrease, and the standard deviation will tend toward zero. For each precision level, we compute the average rolling standard deviation across all 50 pairs. Fig B shows the evolution of this average standard deviation, providing a visual indicator of convergence behavior across pairs.

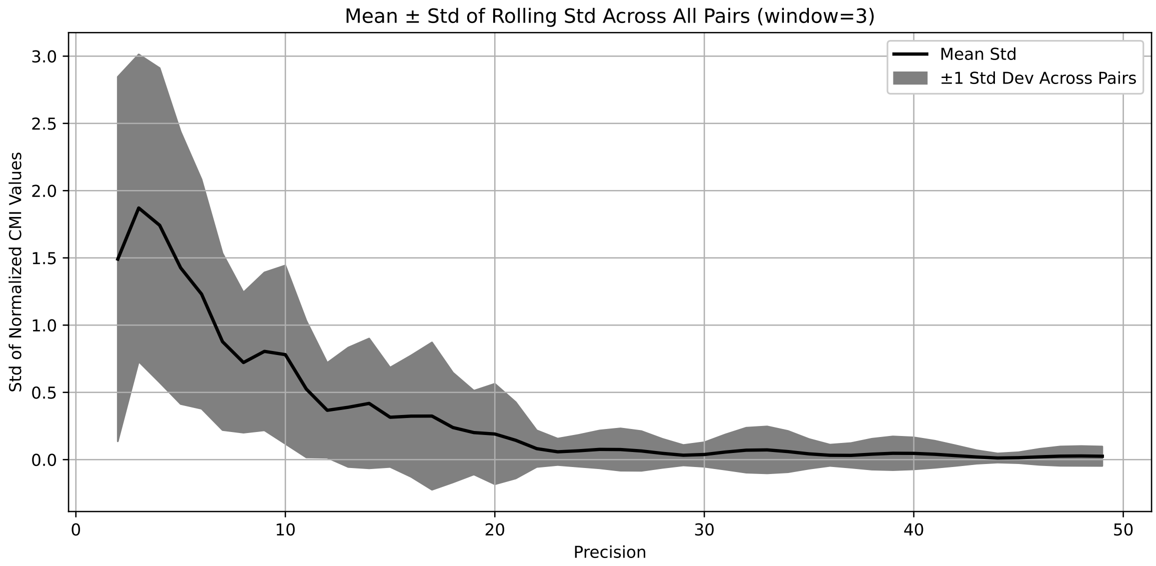

Fig B: CMI Standard Deviation. Evolution of the mean standard deviation of the estimated CMI across 50 different interactions in different values of precision. The value is calculated using a rolling window of 3 precisions. It is visible how this value tends to zero after 20 trapezoid, showing a mostly complete convergence in CMI estimation. The shaded area represents the standard deviation of the mean standard deviation values across the 50 different interactions.

From approximately 23–24 trapezoids onward, the standard deviation stabilizes near zero, indicating that most pairs have likely converged to their true value at this point. Since these values represent the standard deviation over the previous three precision levels, a default value of 20 trapezoids has been chosen. This value provides a reliable estimation, ensuring convergence in virtually all cases. Users may reduce this precision to gain efficiency at the expense of accuracy. Although the exact execution time depends on the processor's characteristics, Fig C suggests a possibly exponential growth in computation time, increasing from approximately 0.25 seconds per iteration at around 10 trapezoids, to $\sim$ 0.5 seconds at 20, $\sim$ 1 second at 30, and $\sim$ 2 seconds at 40. Therefore, it is advisable to use the lowest precision values possible.

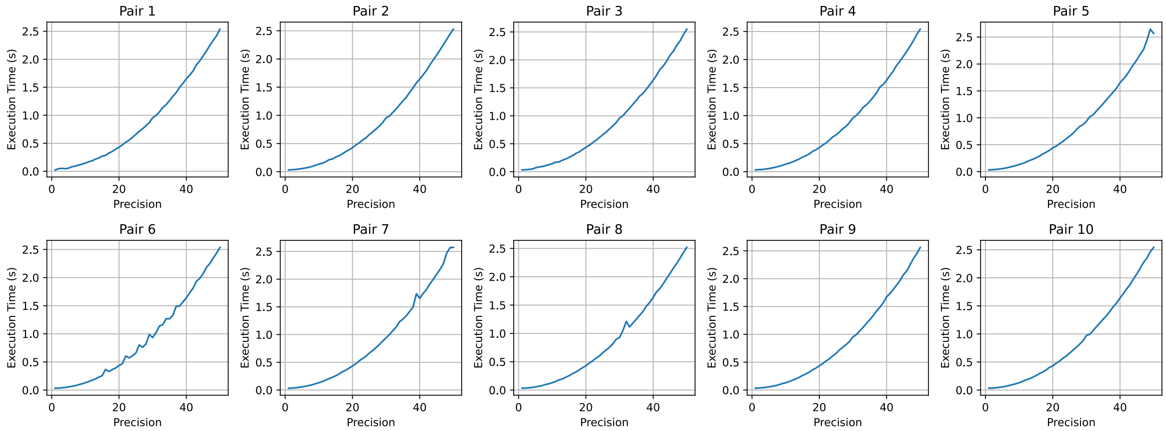

Fig C: CMI execution time. Evolution of the execution times for *CMI* estimation with different values for precision.

Interaction hierarchy

The interaction hierarchy determines the order in which interactions are incorporated into the network topology. This ranking is built by taking into account the interaction scores. Four different methods are available and can be manually selected by user: *'alternative'*, *'structural'*, *'functional'* and *'weighted'*. By default, RNACOREX implements the *'alternative'* method.

- **Alternative**

It is the original ranking method of RNACOREX and the one implemented by default by the model. In this approach, interactions are separately ranked by their structural and functional information scores and alternatively included in the final interaction ranking. Every interaction will be present in both structural and functional rankings, and consequently, once an interaction is added to the final ranking, other occurrences of this interaction are ignored.

- **Structural / Functional**

Structural and functional methods allow user to build the networks using only structural or functional information scores, respectively. When selecting one of this methods, only structural or functional rankings are considered, and this is set as the final interaction ranking, selecting interactions by their appearance order in this ranking.

- **Weighted**

The weighted method calculates a weighted score using both structural and functional scores for each interaction. This weighted score is simply a weighted mean between scores, using a tuning parameter λ controlling the weight given to the structural information score. λ will be in the [0,1] range and can be manually selected by the user defining the *'weight'* parameter along with the *'weighted'* mode.

$$w_{ij} = SS_{ij} \cdot \lambda + FS_{ij} \cdot (1 - \lambda) \tag{9}$$

Tie-breaking procedures

During the construction of the interaction ranking, it is possible for two or more interactions to obtain identical values for their structural or functional information scores. These situations, referred to as ties, can have a considerable impact on the resulting network topology, since the relative ordering of tied interactions determines which edges are included at different ranking thresholds. It is therefore important to define an appropriate tie-resolution strategy that aligns with the user's analytical goals.

In practice, most ties arise from the structural score, which depends only on the set of databases (or prediction engines) in which an interaction appears. Interactions present in exactly the same databases will naturally receive identical structural scores. By contrast, the functional information score is calculated from conditional mutual information, getting a more wide and continuous range of values that rarely produce ties.

Three complementary strategies are implemented. The default *'isolated'* method prioritizes interactions between less connected nodes by summing the degrees (total incoming and outgoing links) of both nodes across all potential interactions. Interactions with lower-summed degrees are ranked higher, which favors more specific or isolated relationships. The *'connected'* method applies the same degree calculation but ranks higher-degree pairs first, prioritizing highly connected or interactions instead of isolated ones. Finally, the *'functional'* method, rather than relying on node connectivity, resolves the ties in the structural score by comparing the functional information scores of the tied interactions. Interactions with higher functional scores are ranked higher, reflecting greater functional relevance while maintaining identical structural importance. The default method tends to produce more distributed networks, generally capturing a larger number of regulatory pathways. By contrast, the *'connected'* method favors elements that dominate many interactions, making it easier to identify a small set of highly relevant elements, but often at the cost of excluding some isolated regulatory pathways. The *'functional'* method provides a balanced approach, lying between these two extremes. In Fig D three networks trained with the same data but different tie-breaking strategies are presented.

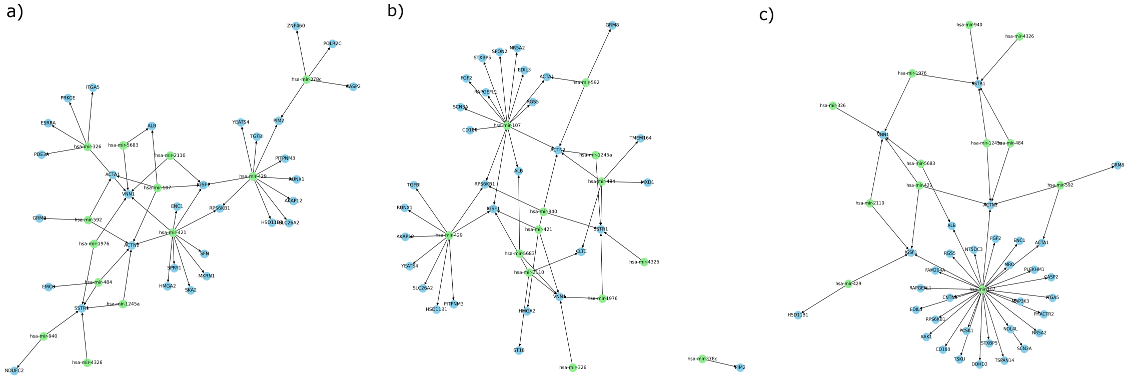

Fig D: Tie-breaking procedures. Three networks with the same number of interactions are shown, constructed using the same input data (BRCA dataset) and different tie-breaking methods: **a) Isolated method. b) Functional method. c) Connected method.**

Conditional Linear Gaussian Classifier

A set of nodes V and a set of edges A, defined as a directed acyclic graph (DAG) $G = (V, A)$, along with a set of parameters θ representing the probability distribution of the nodes, fully describe a Bayesian network $B = (G, \theta)$. In a Bayesian network, the parents of a node D-separate it from the rest of the network, making the node conditionally independent of all other nodes given its parents pa_i. Thanks to these conditional independence relationships, the joint distribution over a set of nodes $(X_1, X_2, ..., X_n)$ can be efficiently computed by considering only the parents of each node $P(\mathbf{X}) = \prod_{X \in \mathbf{X}} P(X|\mathbf{pa_x})$.

Conditional Linear Gaussian models (CLGs) are specific Bayesian Networks encoding both continuous and discrete variables. In these models, continuous variables are encoded with Conditional Linear Gaussian densities and are forced to have only continuous descendants. Being $\mathbf{X} = \{\mathbf{X_D}, \mathbf{X_C}\}$ the set of discrete $\mathbf{X_D}$ and continuous $\mathbf{X_C}$ variables of a CLG, a continuous variable $X_i \in \mathbf{X_C}$ can be part of two type of dependencies: only discrete parents or discrete and continuous parents. If X_i has only discrete parents $\mathbf{pa_i} = \{\mathbf{C}\}$ with $\mathbf{C} \in \mathbf{X_D}$, its distribution is defined as a mixture of Gaussians, with one Gaussian per combination of the discrete parent values $P(X_i|\mathbf{pa_i}) = P(X_i|\mathbf{C}) \sim N(\mu(\mathbf{C}), \sigma^2(\mathbf{C}))$. If, apart from the discrete parents, it is also descendant of continuous nodes $\mathbf{pa_i} = \{\mathbf{Y}, \mathbf{C}\}$, with $\mathbf{C} \in \mathbf{X_D}$ and $\mathbf{Y} \in \mathbf{X_C}$, X_i is modeled using a conditional linear Gaussian distribution, where the mean is a linear function of the continuous parents $P(X_i|\mathbf{pa_i}) = P(X|\mathbf{Y}, \mathbf{C}) \sim N(\beta_0(\mathbf{C}) + \beta^T(\mathbf{C}) \cdot \mathbf{Y}, \sigma^2(\mathbf{C}))$. A Conditional Linear Gaussian classifier is a special case of this model where a single discrete variable C acts as the parent of all other variables in the network. In this configuration, the model defines a conditional distribution $P(\mathbf{X}|C)$, which can be used in conjunction with Bayes' theorem to compute the posterior $P(C|\mathbf{X})$, effectively allowing the model to classify the value of C given observed evidence in the remaining variables. This structure naturally aligns with the requirements of RNACOREX, where a a binary class (C) is classified by using a set of continuous variables (miRNAs and mRNAs). Post-transcriptional coregulation networks add some specific constraints, as only miRNA-mRNA dependencies are allowed. This maps the fact that miRNAs bind to mRNAs to regulate their activity but not viceversa.

The topology of the network will be defined by using the interaction hierarchy. For a k number of interactions, the network will be composed of the first k interactions in the hierarchy. The class C will be defined as predecessor of all the introduced elements. Once the network structure is defined and the dependencies are known, the parameters of each conditional linear Gaussian distribution can be estimated from data. For a $X_i \in \mathbf{X_C}$ node with continuous parents $\mathbf{pa_i} = \{\mathbf{Y}, C\}$, let $\mathbf{Z} = \{X_i, \mathbf{Y}\}$ be the set of elements composed of the node under study and its continuous parents. The joint distribution of $\mathbf{Z}$ can be defined using the n-dimensional mean vector μ and the n x n dimensional covariance matrix Σ, $P(\mathbf{Z}) \sim N(\mathbf{Z}; \mu; \mathbf{\Sigma})$. The mean vector and the covariance matrix of the joint distribution are used to estimate the parameters of X as a function of its parents $P(X|\mathbf{pa_X}) = P(X|\mathbf{Y}, C) \sim N(\beta_0(C) + \beta^T(C) \cdot \mathbf{Y}, \sigma^2(C))$.

$$\beta_{0_{X|\mathbf{Y},C}} = \mu_{X|C} - \Sigma_{X\mathbf{Y}|C}\Sigma_{\mathbf{Y}|C}^{-1}\mu_{\mathbf{Y}|C}$$

$$\boldsymbol{\beta}_{X|\mathbf{Y},C} = \Sigma_{\mathbf{Y}|C}^{-1}\Sigma_{\mathbf{Y}X|C}$$

$$\sigma_{X|\mathbf{Y},C}^2 = \Sigma_{X|C} - \Sigma_{X\mathbf{Y}|C}\Sigma_{\mathbf{Y}|C}^{-1}\Sigma_{\mathbf{Y}X|C}$$

For new evidence in the continuous nodes $\mathbf{X_R}$, the CLG gives a posterior probability associated to each of the classes encoded by the model $c \in \Omega_C$.

$$P(C|\mathbf{X_R}) = \frac{P(C, \mathbf{X_R})}{P(\mathbf{X_R})} = P(C) \cdot \frac{P(\mathbf{X_R}|C)}{P(\mathbf{X_R})} \tag{10}$$

The class with the highest assigned posterior probability c^* is then selected as the predicted class.

$$c^* = argmax_{c\in\Omega_C} P(c) \cdot \prod_{X_i \in X_R} P(X_i|\mathbf{Y_i}, c), \tag{11}$$

References

[1] Armañanzas R, Saeys Y, Inza I, García-Torres M, Bielza C, van de Peer Y, Larrañaga P. Peakbin selection in mass spectrometry data using a consensus approach with estimation of distribution algorithms. *IEEE/ACM Trans Comput Biol Bioinform.* 2011 May-Jun; 8(3): 760-774.

[2] Kuncheva LI. A Stability Index for Feature Selection. Proc. 25th *IASTED Int'l Multi-Conf. Artificial Intelligence and Applications.* 2007, pp. 390-395.

[3] Cover TM, Thomas JA. Elements of information theory. *John Wiley & Sons.* Ltd. pp. 13–55, 2005.

[4] Parzen E. On estimation of a probability density function and mode. *The Annals of Mathematical Statistics.* 1962 33(3): pp. 1065–1076.

[5] Chen YC. A tutorial on kernel density estimation and recent advances. *Biostatistics & Epidemiology*, 2017 1(1), pp. 161-187.

[6] Scott DW. Multivariate Density Estimation: Theory, Practice, and Visualization. *John Wiley & Sons.* New York, Chicester, 1992.

[7] Turlach BA. Bandwidth Selection in Kernel Density Estimation: A Review. *CORE and Institut de Statistique.* 1993, Vol. 19, pp. 1-33.

[8] Jones MC, Marron JS, Sheather SJ. A brief survey of bandwidth selection for density estimation. *Journal of the American Statistical Association.* 2015 Mar; 91 (433); pp. 401-407

[9] Silverman BW. Density estimation for Statistics and Data Analysis. *Monographs on Statistics and Applied Probability.* 1986, London: Chapman and Hall.

[10] Virtanen P, Gommers R, Oliphant TE, Haberland M, Reddy T, Cournapeau D, et al. SciPy 1.0: Fundamental Algorithms for Scientific Computing in Python. *Nature Methods.* 2020, Vol. 17, pp. 261–272.

[11] Burden RL, Faires JD. Numerical Analysis. *Boston: PWS-Kent Publishing Company.* 1989.
