# Supplementary material for: RNACOREX - RNA coregulatory network explorer and classifier
Source: PLoS Comput Biol. 2025 Nov 3;21(11):e1013660. doi: 10.1371/journal.pcbi.1013660 (PMC12594346; doi:10.1371/journal.pcbi.1013660)
Supplement: S3 Text — (PDF) [file pcbi.1013660.s003.pdf]

Package implementation

Availability

RNACOREX package is open-source with an APACHE 2.0 license. It is available in different repositories:

- **Github**. Code, package versions, article code, data and quick-start examples are accessible at: `https://github.com/digital-medicine-research-group-UNAV/RNACOREX`.
- **PyPI**. Package is also available on the Python Package Index (PyPI) at: `https://pypi.org/project/rnacorex/`
  It allows direct installation via `pip install rnacorex`. Reading package documentation is highly recommended before running RNACOREX.
- **Zenodo**. A Docker image of the package including all necessary dependencies for running is also available at Zenodo under the DOI '10.5281/zenodo.17397953'.

Prerequisites

To operate correctly, RNACOREX relies on external databases that provide essential structural and functional interaction information. Specifically, the package requires data from DIANA microT, TargetScan, miRTarBase, TarBase, and GENCODE. Due to size limitations, these databases are not included in the package installation via PyPI, nor are they hosted in the GitHub repository. As a result, users must download these resources after package installation. Users can run the built-in function `download()` to authomatically download all databases. These databases will be saved in the '/engines' directory within the package structure. The `check_engines()` function can be used to verify that all required resources have been correctly downloaded. If the function returns a positive result, the package is ready for execution. If there is any problem with the download, the databases can be manually downloaded from the following URL: `https://tinyurl.com/RNACOREX`. The databases should be placed inside the '/engines' folder within the package directory structure.

It is important to note that in the Docker image distributed via Zenodo, this function is not needed, as the image already includes all necessary databases. In that case, RNACOREX is preconfigured and will run automatically without requiring any additional setup by the user.

RNACOREX requires the following dependencies, which are automatically installed when the package is executed via Zenodo or PyPI.

- `Python` 3.9 +
- `matplotlib` 3.8.4 +
- `networkx` 3.1 +
- `numpy` 2.0.0 +
- `pandas` 2.1.4 +
- `scipy` 1.13.1 +
- `scikit-learn` 1.4.2 +
- `tqdm` 4.65.0 +
- `joblib` 1.5.1 +

`pygraphviz` (version 1.9 or higher) is also required for visualizing post-transcriptional networks. This dependency is included when running the Docker image available on Zenodo. However, it is not automatically installed when the package is installed via `pip`. In the latter case, to avoid dependency issues, it is strongly recommended to install `pygraphviz` via `conda` using the following command: '`conda install -y -c conda-forge pygraphviz`'. When installing the package via `pip`, `gdown` is also authomatically installed in order to download the required engines. This dependency is not necessary when running it in Zenodo as the databases are already downloaded.

MRNC

MRNC is the main class implemented in RNACOREX. A MRNC object has five main attributes: n_con, precision, mode, weight and ties. The n_con value corresponds with the default number of connections used for fitting the model and displaying the network, precision will set the precision of the trapezoidal method in the KDE estimation and mode will define the method for computing the interaction ranking. Weight is only required if mode = 'weighted' is selected and will represent the value of the λ parameter, defining the given weight to the *structural information scores.* Ties will define the tie-breaking procedure. All values are optional and can be modified by the user.

The MRNC object is initialized with a X and y databases which should meet certain characteristics. The X database is associated to the mRNA and miRNA expression data, being composed of $n + m$ columns, with n being the number of miRNAs and m the number of mRNAs. The quantification method for these expressions is not relevant in here, but it must be consistent, i.e., it should be the same for all miRNAs and mRNAs. Ideally, and for a more efficient running, it is advised to include only differentially expressed mRNAs. For mRNAs, the model works with Ensembl identifiers [1], i.e. *'ENSG00000004139'*, this way, all genomic elements with a code not starting by *'ENSG'* will be removed. The microRNA data is managed with miRBase identifiers, i.e. *'hsa-let-7c'*. The HGNC (Human Genome Organization Gene Nomenclature Committee) [2] or 'Hugo Symbol' nomenclature is also used for displaying mRNAs in the resulting network but it is not allowed in the input database. It is important to follow this instructions and use these nomenclatures in the input database. The y database will correspond to the phenotype or the class with a unique binary column, encoded with 0 or 1 values.

Some of the most useful methods implemented by RNACOREX are briefly explained below. Not all methods are required to develop the main RNACOREX pipeline, but their implementation enables specific extensions of the package's functionality. Optional arguments are marked with a star (*).

- fit(X_train, y_train) - Directly implements the full RNACOREX pipeline, initializes the model, computes structural and functional information and estimates the parameters of the CLG at once.

- predict(X_test) - Uses a trained model from fit() and makes the classification for a test set.

- predict_proba(X_test) - Makes the classification for a test set as in predict() but also returns the class probability for each sample.

- get_network(k*, display*) - The interaction hierarchy is used to load the coregulation network with k interactions. If k is not specified, the network containing n_con interactions is loaded by default. The network is returned as a networkx object and stored in mrnc.G_. If display = True is set, the network is also visualized.

- initialize_model(X_train, y_train) - Initializes the set of miRNAs and mRNAs by combining the input database with expert and validated knowledge extracted from *TargetScan*, *Diana micro-T* and *miRTarBase*. It also calculates the structural information contained in each miRNA-mRNA interaction.

- compute_functional(X_train*, y_train*) - Uses the trapezoidal rule to estimate the conditional mutual information of every miRNA-mRNA-class triplet. Implements a Gaussian kernel and the Scott's approximation of the normal reference rule as bandwidth method by default. The precision of the trapezoidal rule is initially set to 20. This parameters could be personalized. The conditional mutual information is computed by default with the train sets used in the model initialization. New train set can be specified if same variables as the ones used for model initialization are respected.

- rank() - Ranks the interactions of the model based on the structural and functional information using the ranking mode defined in the MRNC class object.

- fit_only(new_sets*) - Estimates the parameters of an individual CLG with k interactions if the model have been previously initialized and the functional information computed. If the model is initialized with a train set and the functional information computed with a different one, new_sets option has to be selected.

As `MRNC` is a `scikit-learn` [3] compatible estimator, it allows the implementation of any other method from that package.

References

[1] Harrison PW, Amode MR, Austine-Orimoloye O, Azov AG, Barba M, Barnes I, et al. Ensembl 2024 *Nucleic Acids Research.* January 2024, Vol. 52, Issue D1, pp. D891–D899.

[2] Bruford EA, Braschi B, Denny P. HGNC: a central resource for human gene nomenclature. *Nucleic Acids Research.* 2021.

[3] Pedregosa F, Varoquaux G, Gramfort A, Michel V, Thirion B. Scikit-learn: Machine Learning in Python. *Journal of Machine Learning Research.* 2011, Vol. 12, pp. 2825-2830.
