# Supplementary material for: RNACOREX - RNA coregulatory network explorer and classifier
Source: PLoS Comput Biol. 2025 Nov 3;21(11):e1013660. doi: 10.1371/journal.pcbi.1013660 (PMC12594346; doi:10.1371/journal.pcbi.1013660)
Supplement: S4 Text — Fig A. Class Assignment.​ Table A. Dataset characteristics.​ (PDF) [file pcbi.1013660.s004.pdf]

Experiments

In the experimentation, RNACOREX is used in order to find the post-transcriptional coregulation network associated to patients with short and long survival in different cancers. This experiments are developed in order to check the model performance and validate the package usability.

Data

Data from TCGA studies is used [1]. TCGA studies are large-scale research projects that aim to improve the understanding of cancer at molecular level. In this studies wide genomic profiling of different cancers is developed, including measuring mRNA and miRNA expression. Data of different TCGA studies is accessed through NCI's Genomic Data Commons (GDC) platform using UCSC Xena portal (Accession: Jan,2024) [2,3]. For the experiments of the paper, data from Breast Invasive Carcinoma (BRCA), Colon Adenocarcinoma (COAD), Head-Neck Squamous Carcinoma (HNSC), Kidney Renal Cell Carcinoma (KIRC), Acute Myeloid Leukemia (LAML), Liver Hepatocellular Carcinoma (LIHC), Lung Adenocarcinoma (LUAD), Lung Squamous Cell Carcinoma (LUSC), Low Grade Glioma (LGG), Sarcoma (SARC), Skin Cutaneous Melanoma (SKCM), Stomach Adenocarcinoma (STAD) and Uterine Corpus Endometrial Carcinoma (UCEC) is downloaded and analyzed.

Preprocessing

TCGA covers many different information, but for the implementation of RNACOREX only survival data and miRNA and mRNA expression is used. Samples include a barcode that identifies the sample type: tumoral (00–09), peritumoral (10–19), or normal/control (20–29), even so, normal/control samples are usually unavailable. As the idea of the experimentation is trying to understand the differences between short-survival and long-survival patients, normal and control samples are not required and therefore removed for the analysis. Therefore, only TCGA sample codes between (01-09) are retained.

In mRNA expression database, transcripts are identified with the Ensembl nomenclature, i.e. *'ENSG00000004139.2'*. This nomenclature represents the mRNA identifier *'ENSG00000004139'* and its annotation version *'.2'*. For the analysis, only elements codified with codes starting with *'ENSG'* are selected. In miRNA expression database, elements are codified using their mirBase nomenclature, i.e. *'hsa-let-7c'*. miRNA, mRNA and survival databases are then joined maintaining only those samples in which both mRNA and miRNA expression data is present. When the three databases are merged, mRNAs and miRNAs with less than 5 and 1 counts in more than 25% of the samples, respectively, are removed.

The class is assigned to each sample depending on his survival time information. The survival database contains two important columns *'OS'* and *'OS.time'*. The first one defines censored and uncensored data, identifying patients who died during the study (uncensored) and patients who don't (censored). The second one shows the time (in days) from the start of the study to the patient's death or the end of the follow-up. If the idea is assigning the class depending on survival time, it would make sense working only with uncensored data, where the survival time is known, and discarding the rest. Even so, more samples could be included by using those censored whose survival time until the end of follow-up exceeds the threshold that defines the boundary between both classes. Using this approach, classes are assigned as shown in Fig A.

When the classes are assigned, differential gene expression (DGE) is developed. DGE is only implemented in mRNAs in order to reduce dimensionality of data and select only relevant elements. It is not developed in miRNAs as the number of this elements in the initial database is much smaller. Differentially expressed genes (DEGs) were identified using the DESeq2 framework as implemented in PyDESeq2 [4]. Raw count data were modeled using a negative binomial distribution to account for biological and technical variability. For each gene, a generalized linear model (GLM) was fitted, relating gene expression to the experimental condition (e.g., class A vs. class B) with the logarithm of expected counts expressed as a linear combination of sample-specific size factors and the condition effect. Hypothesis testing was performed using the Wald test to evaluate whether the estimated log2 fold change between conditions was significantly different from zero. The resulting p-values were adjusted for multiple testing using the Benjamini-Hochberg procedure to control the false discovery rate (FDR).

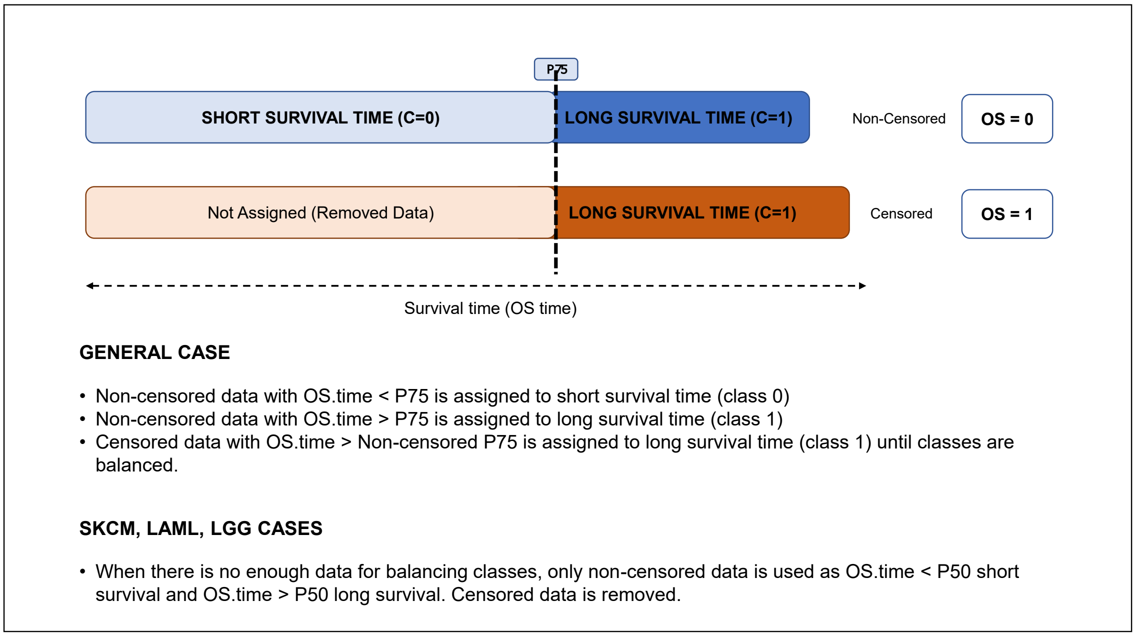

Fig A: Class assignement. The 75% of the uncensored samples with lower survival time are assigned to the short survival time class. In the other class, the long survival time samples are stored, including the rest of the uncensored samples (the 25% with longest survival time) and censored samples with a follow-up time of at least the value located in the boundary between classes. In some cases, there is not enough censored data to complete the long survival class as their follow-up times are short or there are few samples. This prevents from obtaining two 50-50 balanced classes. In those cases (*SKCM*, *LAML* and *LGG*), only uncensored samples are used.

Genes with an adjusted p-value (FDR) below 0.05 were considered significantly differentially expressed and retained for further analysis.

The database with the raw counts is filtered, selecting only the differentially expressed genes identified by PyDeSeq2. This count values are then log-normalized using a $log_2(x+1)$ normalization. With the clean and filtered log-normalized database, the Ensembl nomenclature of each gene is again adapted removing the version number of the code, represented after the dot (i.e *ENSG00000139618.11* to *ENSG00000139618*). With this last change, the final database is obtained. As an example, in Table A the starting characteristics of the final databases are represented.

Model Specifications

- **RNACOREX**

Networks with different number of interactions are analyzed in order to find the network which best fits the data. Starting from the most simple network with a single interaction, 200 networks are built for each database, sequentially increasing the complexity of the network by adding a new interaction. Therefore, networks from 1 to 200 interactions are executed. Interaction ranking is built with the *"alternative"* mode, selecting alternatively interactions from structural and functional rankings. The by default tie-breaking strategy *"isolated"* is selected, including most isolated interactions in case of ties. The model is trained using a stratified three fold cross-validation (CV) framework, implementing three iterations for each network with a different balanced train-test split. The precision of the functional information is set to 20.

- **Other models**

For each value of k, five additional classification models are fitted in order to compare their performance with the CLG. Among these five models, three are traditional vector-based classifiers

Table A. Dataset Characteristics

Disease	Samples	miRNA input	mRNA input	miRNA model	mRNA model	Interactions
BRCA	224	297	950	173	756	2810
COAD	140	302	125	172	102	314
HNSC	324	310	2326	177	2019	9679
KIRC	250	275	6484	156	4993	17945
LAML	77	304	837	164	687	3696
LIHC	191	286	1603	167	1408	4567
LUAD	262	294	2187	170	1873	6356
LUSC	280	318	809	185	672	3095
LGG	135	301	6932	166	5519	23447
SARC	146	260	998	144	841	2135
SKCM	213	346	4055	185	3716	18571
STAD	216	290	1058	167	846	2446
UCEC	131	314	2643	181	2326	11605

Table notes. This table shows the information and characteristics of the databases used in the experimentation. **Samples** stands for the number of patients in the database, divided as 50-50 between classes. **Input data** and **model data** show the number of miRNAs and mRNAs in the initial database (after DGE) and after filtering with the validated databases, respectively. **Interactions** represents the number of miRNA-mRNA connections included in the model.

(Random Forest, SVM, and Gradient Boosting), while the other two are specifically designed for graph classification tasks (GNN and Graph Kernel). For a fair competition, models are fitted with the same train-test split in each iteration, using only the information of the nodes present in the CLG.

Vector-based classifiers. Random Forest, SVC and Gradient Boosting classifiers are built mainly with *scikit-learn* default characteristics. Specific parameters are 100 estimators for Random Forest, 'rbf' kernel in SVC and 20 estimators and a learning rate of 0.2 in Gradient Boosting.

Graph Neural Network (GNN). A GNN model is implemented for graph classification tasks. In this setup, each data sample during training is represented by an individual graph, and the model learns to classify these samples by exploiting their corresponding graph structure. For every value of k, all samples share a common underlying graph topology extracted from RNACOREX, while the node features are defined solely by expression data. The graph convolution process is carried out using two Graph Convolutional Network (GCN) layers with input and output feature dimensions of (1,16) and (16,32), respectively. Each GCN layer is followed by a ReLU activation function. After the second GCN layer, a global mean pooling operation is applied to aggregate node features into a graph-level representation for each sample in a batch of size 16. The pooled representation is passed through a fully connected layer to produce the logits corresponding to each class. Finally, a log-softmax function is applied to obtain log-probabilities for classification. Model training is performed using the Adam optimizer with a learning rate of 0.01, and the Negative Log Likelihood Loss (NLLLoss) is used as the loss function to guide the optimization process.

Graph Kernel (GK). Implements a graph kernel-based classification approach using the Weisfeiler-Lehman (WL) subtree kernel combined with a Support Vector Machine (SVM) classifier. Each sample is converted to a graph using the graph structure as extracted from RNACOREX. A binary label is assigned to each node by comparing the expression value in the sample against the median of that feature across all training samples. WL kernel computes similarity matrices between the graphs of the train and test samples using three iterations. A Support Vector Machine (SVM) with the WL precomputed kernel and a $C = 1$ regularization parameter is trained on the training kernel matrix.

References

[1] Tomczak K, Czerwińska P, Wiznerowicz M. The Cancer Genome Atlas (TCGA): an immeasurable source of knowledge. *Contemp Oncol (Pozn).* 2015; 19(1A): A68-77.

[2] Grossman R, Heath A, Ferretti V, Varmus HE, Lowy DR, Kibbe WA. Toward a Shared Vision for Cancer Genomic Data. *The New England Journal of Medicine.* 2016.

[3] Goldman M, Craft B, Hastie M, Repečka K, McDade F, Kamath A, et al. The UCSC Xena platform for public and private cancer genomics data visualization and interpretation. *bioRxiv.* 326470, 2019.

[4] Muzellec, B., et. al. PyDESeq2: a python package for bulk RNA-seq differential expression analysis *Bioinformatics.* 2023 Sept; 39(9).
