# Supplementary material for: RNACOREX - RNA coregulatory network explorer and classifier
Source: PLoS Comput Biol. 2025 Nov 3;21(11):e1013660. doi: 10.1371/journal.pcbi.1013660 (PMC12594346; doi:10.1371/journal.pcbi.1013660)
Supplement: S5 Text — Table A. Performance.​ Fig A. Most Repeated RNAs.​ Fig B. Pancancer Interactions.​ Fig C. Shared and tissue-specific interactions.​ Table B. Interaction ranking.​ Fig D. miR-1293 interactions in HNSC.​ Fig E. Gene expression levels.​ Fig F. miR-1293 interacting gene metrics.​ (PDF) [file pcbi.1013660.s005.pdf]

Results

Model Performance

Table A shows the performance of RNACOREX in the 13 databases from the TCGA that have been analyzed. Results show the performance metrics (Accuracy and AUC) obtained by RNACOREX along with the metrics of the other 5 models. RNACOREX obtains accuracy metrics ranging 0.59 and 0.75 for the best network and 0.57 and 0.71 for the main accuracy in all networks. Maximum AUC stays in the 0.64-0.80 range and mean AUC goes from 0.62 to 0.77. These results are influenced by the fact that the structure search was conducted with a limitation of 200 interactions. Allowing the development of models with a greater number of interactions could result in optimal networks with more edges and better accuracy values. Other graph-based classification models like GNN and Graph Kernel, appear to fail on catching class-specific patterns in this specific problem, as their metrics are clearly worse than with other models. Vector-based classification algorithms like Random Forest, SVM and Gradient Boosting seem to work better, showing similar accuracy metrics when comparing to RNACOREX. However, RNACOREX offers a significant advantage: its results are fully explainable and the associated coregulation network can be easily extracted, identifying which MTI's are affecting the class under study.

Table A. Performance

(A) RNACOREX, GNN, and GKER

Dataset	**RNACOREX**				**GNN**				**GKER**			
	Max. Acc	Max. AUC	Mean Acc.	Mean AUC	Max. Acc	Max. AUC	Mean Acc.	Mean AUC	Max. Acc	Max. AUC	Mean Acc.	Mean AUC
BRCA	0.68	0.69	0.64 (0.02)	0.67 (0.02)	0.59	0.60	0.53 (0.03)	0.53 (0.03)	0.58	0.57	0.52 (0.03)	0.49 (0.03)
COAD	0.66	0.70	0.63 (0.02)	0.67 (0.02)	0.62	0.63	0.53 (0.03)	0.54 (0.03)	0.60	0.62	0.49 (0.04)	0.48 (0.05)
HNSC	0.65	0.67	0.62 (0.02)	0.66 (0.02)	0.57	0.59	0.51 (0.02)	0.53 (0.02)	0.61	0.64	0.52 (0.03)	0.52 (0.03)
KIRC	0.60	0.64	0.57 (0.02)	0.62 (0.02)	0.63	0.63	0.57 (0.03)	0.59 (0.03)	0.63	0.66	0.56 (0.03)	0.56 (0.04)
LAML	0.75	0.80	0.71 (0.03)	0.77 (0.02)	0.69	0.75	0.59 (0.04)	0.61 (0.04)	0.67	0.66	0.55 (0.04)	0.54 (0.05)
LGG	0.68	0.73	0.67 (0.02)	0.72 (0.02)	0.67	0.67	0.62 (0.02)	0.64 (0.01)	0.64	0.67	0.55 (0.03)	0.55 (0.05)
LIHC	0.68	0.71	0.65 (0.02)	0.70 (0.01)	0.61	0.64	0.56 (0.02)	0.59 (0.02)	0.62	0.61	0.55 (0.03)	0.53 (0.05)
LUAD	0.67	0.70	0.64 (0.02)	0.69 (0.02)	0.54	0.54	0.50 (0.02)	0.49 (0.02)	0.59	0.61	0.54 (0.02)	0.53 (0.04)
LUSC	0.63	0.69	0.61 (0.02)	0.66 (0.03)	0.57	0.57	0.54 (0.01)	0.55 (0.01)	0.63	0.60	0.54 (0.03)	0.51 (0.04)
SKCM	0.59	0.64	0.57 (0.01)	0.62 (0.01)	0.59	0.59	0.56 (0.01)	0.57 (0.02)	0.59	0.61	0.53 (0.03)	0.50 (0.04)
SARC	0.70	0.73	0.67 (0.03)	0.71 (0.02)	0.64	0.67	0.58 (0.03)	0.64 (0.02)	0.68	0.69	0.58 (0.03)	0.59 (0.05)
STAD	0.64	0.68	0.61 (0.02)	0.66 (0.01)	0.57	0.55	0.51 (0.02)	0.50 (0.03)	0.60	0.60	0.53 (0.03)	0.52 (0.04)
UCEC	0.72	0.77	0.69 (0.02)	0.74 (0.01)	0.64	0.67	0.57 (0.03)	0.60 (0.05)	0.69	0.68	0.57 (0.04)	0.59 (0.04)

(B) Random Forest, SVM, and Gradient Boosting

Dataset	**Random Forest**				**SVM**				**Gradient Boosting**			
	Max. Acc	Max. AUC	Mean Acc.	Mean AUC	Max. Acc	Max. AUC	Mean Acc.	Mean AUC	Max. Acc	Max. AUC	Mean Acc.	Mean AUC
BRCA	0.69	0.73	0.64 (0.03)	0.69 (0.03)	0.71	0.78	0.68 (0.03)	0.73 (0.05)	0.70	0.72	0.62 (0.03)	0.67 (0.03)
COAD	0.69	0.72	0.63 (0.03)	0.66 (0.03)	0.69	0.69	0.65 (0.03)	0.57 (0.07)	0.66	0.71	0.59 (0.04)	0.62 (0.04)
HNSC	0.64	0.68	0.60 (0.02)	0.64 (0.02)	0.63	0.67	0.61 (0.02)	0.64 (0.03)	0.66	0.66	0.58 (0.03)	0.61 (0.02)
KIRC	0.66	0.70	0.62 (0.02)	0.66 (0.03)	0.70	0.71	0.65 (0.02)	0.70 (0.03)	0.63	0.66	0.58 (0.02)	0.62 (0.02)
LAML	0.71	0.74	0.65 (0.03)	0.69 (0.03)	0.73	0.75	0.69 (0.03)	0.73 (0.04)	0.66	0.69	0.59 (0.04)	0.63 (0.03)
LGG	0.68	0.75	0.63 (0.02)	0.71 (0.02)	0.66	0.75	0.63 (0.02)	0.73 (0.02)	0.68	0.74	0.63 (0.03)	0.69 (0.03)
LIHC	0.70	0.75	0.66 (0.02)	0.71 (0.03)	0.68	0.75	0.66 (0.02)	0.72 (0.03)	0.72	0.75	0.64 (0.03)	0.68 (0.03)
LUAD	0.65	0.68	0.61 (0.02)	0.65 (0.02)	0.64	0.69	0.61 (0.02)	0.61 (0.09)	0.61	0.64	0.57 (0.02)	0.60 (0.02)
LUSC	0.62	0.65	0.57 (0.02)	0.61 (0.02)	0.62	0.66	0.58 (0.02)	0.62 (0.03)	0.59	0.62	0.55 (0.02)	0.58 (0.02)
SKCM	0.68	0.69	0.63 (0.02)	0.64 (0.02)	0.65	0.67	0.63 (0.02)	0.61 (0.07)	0.68	0.66	0.61 (0.03)	0.62 (0.03)
SARC	0.73	0.77	0.67 (0.03)	0.72 (0.04)	0.73	0.77	0.69 (0.03)	0.74 (0.03)	0.71	0.75	0.63 (0.03)	0.68 (0.04)
STAD	0.65	0.68	0.60 (0.02)	0.64 (0.02)	0.61	0.66	0.58 (0.01)	0.64 (0.01)	0.63	0.66	0.58 (0.02)	0.61 (0.02)
UCEC	0.73	0.79	0.68 (0.02)	0.75 (0.02)	0.74	0.81	0.71 (0.02)	0.79 (0.02)	0.75	0.78	0.64 (0.03)	0.70 (0.03)

Table notes. Performance metrics of six models across 13 datasets. **Max. Acc.** and **Max. AUC** are the accuracy and AUC of the best-performing model across k values. **Mean Acc.** and **Mean AUC** show averages across all k values.

TCGA pan-cancer analysis

With the extracted post-transcriptional coregulation network, a brief biological analysis was developed in order to identify the most recurrent elements and interactions. This analysis was also divided into tissue-specific and shared elements. Fig A presents the most repeated elements. While 'hsa-mir-1293' and 'BACH2' appear as the most common tissue-specific miRNAs and mRNAs, 'hsa-mir-378c' and 'SLC2A1' and 'TBL1XR1' were identified as the most repeated elements appearing in more than one tissue. Some bibliographical references, connecting this elements with previous analysis are included in the main manuscript. While the order of appearances remains similar in mRNAs and tissue-specific miRNAs, the number of appearances of non-specific miRNAs is clearly higher. It is important to note that the differential expression analysis performed on mRNAs makes it substantially more difficult for these elements to appear across multiple tissues. In order to be part of the network, an mRNA must first be identified as differentially expressed and then selected as one of the most relevant features. In contrast, miRNAs did not undergo a differential expression filtering step, which means they were considered in all tissues from the start. Additionally, the smaller number of total miRNA elements increases the likelihood that they will be integrated into the network and appear recurrently across tissues. While the order of appearances remains similar in mRNAs and tissue-specific miRNAs, the number of appearances of non-specific miRNAs is clearly higher.

Fig C illustrates the number of tissue-specific and shared interactions across the different databases. The majority of interactions in each database are tissue-specific, ranging from 151 in SARC to just 36 in LUAD. Among tissue pairs, KIRC and SKCM share the highest number of interactions (12), followed by LUAD–SKCM sharing up to 6 interactions. The post-transcriptional network derived for COAD is the most tissue-specific, sharing only two interactions with LAML. In Fig B three interactions appear as the most repeated, with presence in 5 different tissues ('hsa-mir-378c - SERPINE1', 'hsa-mir-378c - SLC2A1' and 'hsa-mir-4326 - TPX2'). This interactions are briefly commented in the main manuscript. Additionally, 3 interactions are shared by at least 4 tissues, and up to 14 interactions are found if interactions shared by 3 tissues are included.

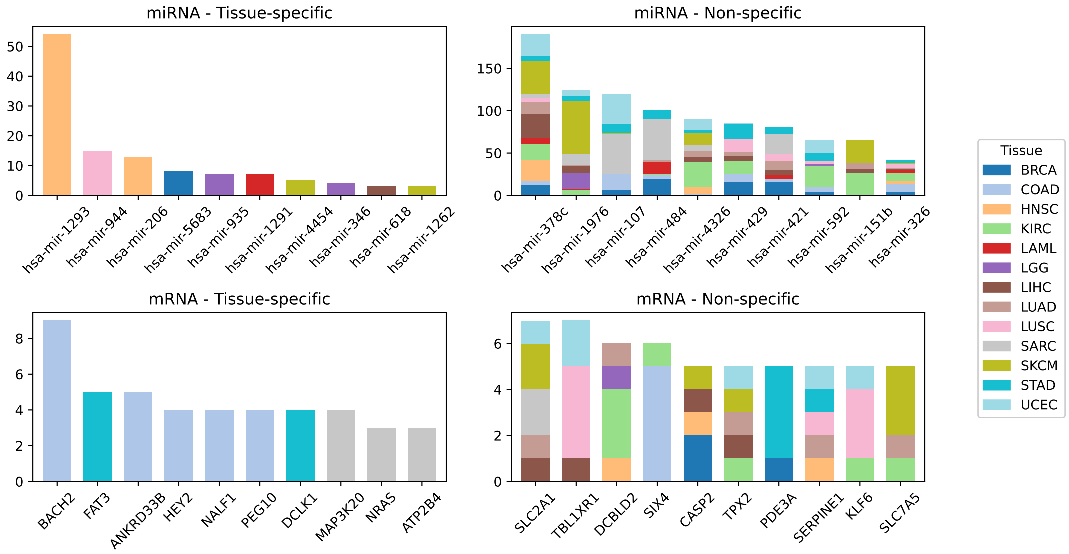

Fig A: Most Repeated RNAs. Number of appearances of the most common mRNAs and miRNAs in the post-transcriptional coregulation networks for both tissue-specific and multicancer scenarios. **(Upper-Left).** Most common tissue-specific miRNAs. **(Upper-Right).** Most common miRNAs appearing in more than one tissue. **(Lower-Left).** Most common tissue-specific mRNAs. **(Lower-Right).** Most common mRNAs appearing in more than one tissue.

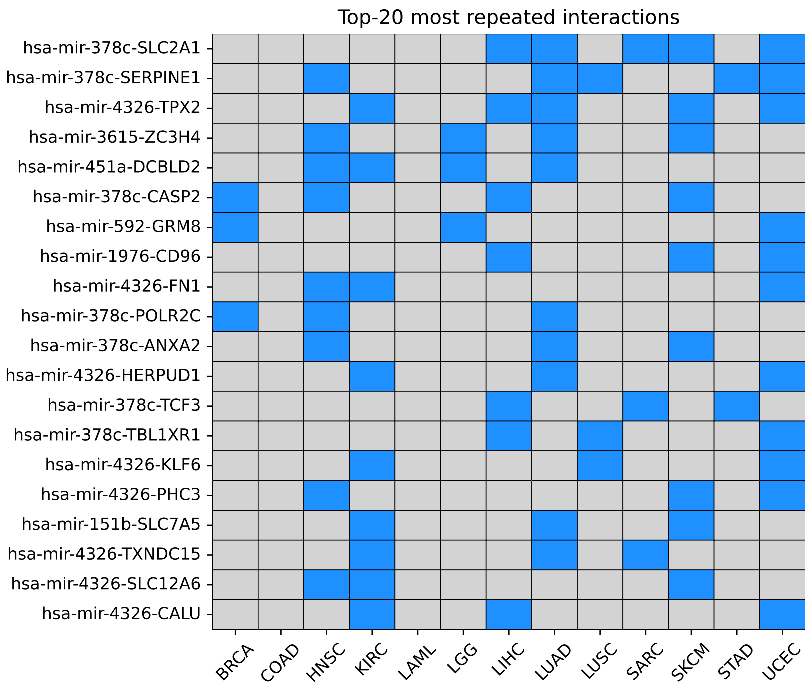

Fig B: Pancancer Interactions. Interactions appearing in at least 3 different tissues ordered by number of tissues.

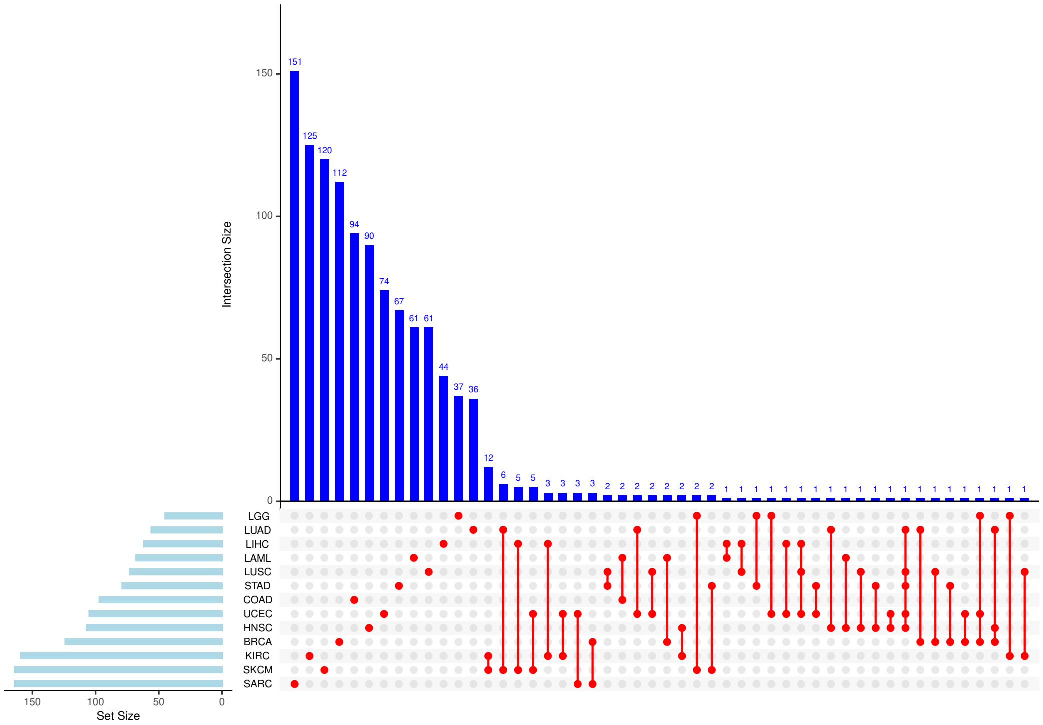

Fig C: Shared and tissue-specific interactions. The figure shows the number of tissue-specific and shared interactions, ordered by appearances.

miR-1293 in Head and Neck Squamous Carcinoma

As an illustrative example, a brief analysis was conducted on some of the most relevant interactions. Fig A shows the most recurrent components across the different networks obtained. Among the tissue-specific miRNAs, miR-1293 stands out, appearing more than 50 times in the HNSC network and representing approximately 50% of that network. Table B presents the top 12 interactions in the HNSC interaction ranking. As can be seen, half of these interactions are associated with this miRNA, particularly those included in the ranking based on functional information (calculated from the expression profiles of the elements). Although only the top 12 interactions are shown in the table, expanding the ranking to the entire network reveals that almost all interactions included through functional information are associated with miR-1293. This observation suggests that the expression of this miRNA and its extensive interactions with multiple genes may have a significant impact on HNSC patients.

Table B. Interaction Ranking.

miRNA	mRNA	SS	FS	cor	p_cor	log2FC	p_adj
hsa-mir-451a	DCBLD2	1.000	0.031	0.035	0.530^{ns}	0.416	0.007^{**}
hsa-mir-1293	RNF150	0.204	0.225	-0.554	0.000^{****}	-0.932	$2.48e-4^{***}$
hsa-mir-7706	CCND1	1.000	0.0389	-0.055	0.316^{ns}	0.445	0.024^{*}
hsa-mir-1293	MCF2L	0.204	0.217	-0.551	0.000^{****}	-0.444	0.025^{*}
hsa-mir-3615	ZC3H4	1.000	0.030	-0.035	0.531^{ns}	-0.140	0.019^{*}
hsa-mir-1293	RHBDL3	0.204	0.193	-0.522	0.000^{****}	-0.970	$2.57e-4^{***}$
hsa-mir-4326	OSBPL11	1.000	0.055	-0.150	0.006^{**}	-0.199	0.009^{**}
hsa-mir-1293	CBX7	0.522	0.190	-0.509	0.000^{****}	-0.425	0.004^{**}
hsa-mir-4326	ARRDC3	1.000	0.039	-0.083	0.134^{ns}	-0.293	0.018^{*}
hsa-mir-1293	ABCA3	0.535	0.187	-0.525	0.000^{****}	-0.936	0.001^{**}
hsa-mir-4326	SLC12A6	1.000	0.049	-0.116	0.035^{*}	-0.296	0.034^{*}
hsa-mir-1293	ZNF775	0.204	0.184	-0.522	0.000^{****}	-0.244	0.048^{*}

Table notes. Main 12 interactions in the interaction ranking for the HNSC network. **miRNA:** Interacting miRNA. **mRNA:** Target mRNA. **SS:** Structural score. **FMI:** Functional score. **cor:** Pearson correlation. **p_cor:** p-value of the correlation. **log2FC:** Log fold change of the classes as calculated by PyDeSeq2. **p_adj:** Adjusted p-value of the log fold change after FDR correction.
*Asterisks indicate statistical significance: (ns) no significative, (*) $p < 0.05$, (**) $p < 0.01$, (***) $p < 0.001$, (****) $p < 0.0001$.

In Fig D, the first six interactions related with miR-1293 are presented. The scatter-plot shows the relation between gene and micro expression, while boxplot shows expression differences between classes. As can be seen in the metrics of the graphics, both the correlation and the class differences are always significative. Additionally, there is a negative correlation in all the cases, showing that when miR-1293 has higher expression, the gene tends to underexpress, aligning with the expected biological behaviour of miRNAs, inhibiting the expression of their target genes.

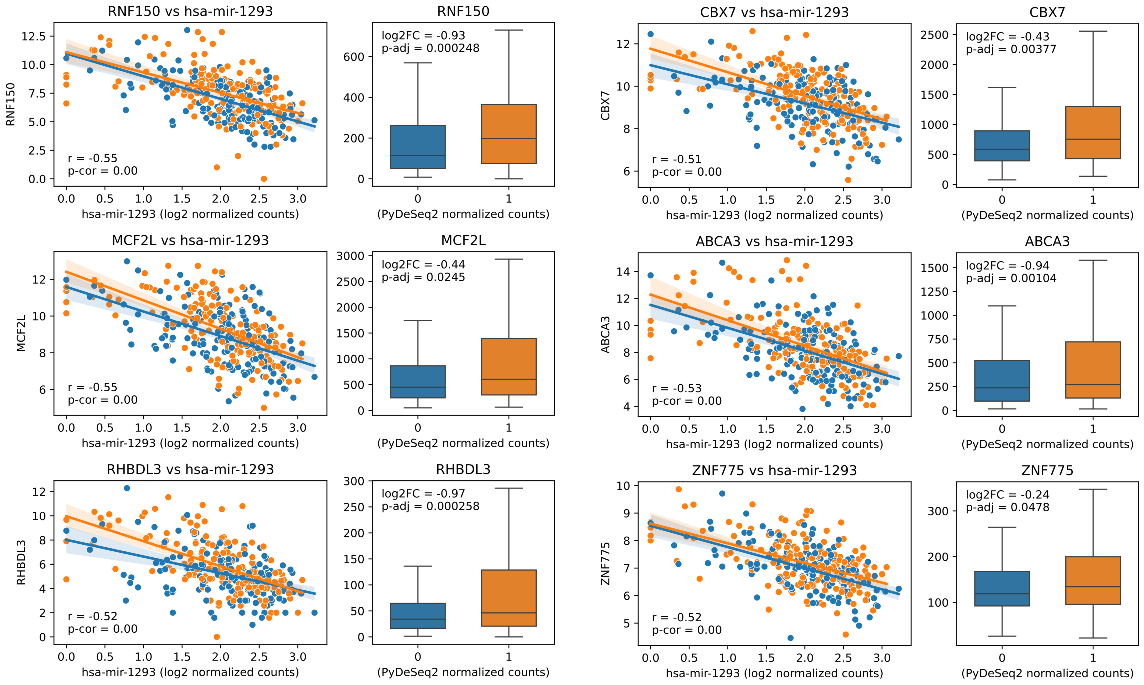

Fig D: miR-1293 interactions in HNSC. The figure shows the main 6 interactions associated with hsa-mir-1293 in HNSC tissue. For each interaction, a scatter plot displays the relationship between the microRNA and gene expression values, and a boxplot shows their distribution by class. The scatter plots use log-normalized counts, while the boxplots show counts normalized as performed by PyDESeq2 during the differential expression analysis.

To gain deeper insight into each interaction, we examined the behavior of the target genes under conditions of low and high expression of the miR-1293. To this end, miRNA expression was dichotomized into two groups of high and low expression using the expression median. As shown in Fig E, gene expression patterns differ markedly between these two conditions, especially in miRNA low expression scenarios. In Table C, some statistics can be accessed. In all cases, the expression of the target gene was higher in the low miRNA expression group, consistent with the negative correlations previously observed. More specifically, when miR-1293 expression was low, the mean expression of the target genes tended to be higher in the long-survival group (Class 1) compared with the short-survival group (Class 0). This difference was statistically significant for MCF2L, RHBDL3, and CBX7, and narrowly missed significance for the remaining three genes. In contrast, under high miRNA expression, differences between survival classes were minimal, except for RNF150, where a small but significant effect persisted.

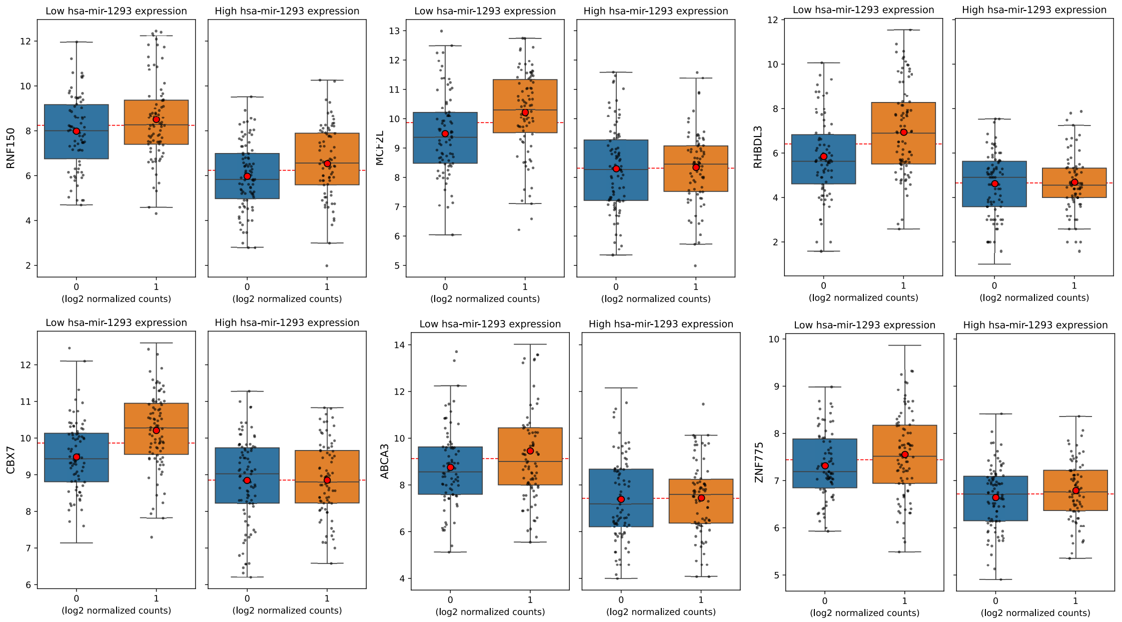

Fig E: Gene expression levels. The figure shows the expression of six genes associated with hsa-miR-1293, stratified by miRNA expression. Samples are divided into low (below median, left panel) and high (above median, right panel) miRNA expression groups. Within each panel, short-survival samples are shown in blue, and long-survival samples in orange. The red dot indicates the mean expression for each class, and the dashed red line indicates the overall mean expression across both classes within the low or high miRNA group.

Table C. miR-1293 interacting gene metrics.

	Low miR-1293				High miR-1293				
**Gene**	μ_0	μ_1	μ_c	p_c	μ_0	μ_1	μ_c	p_c	p_T
RNF150	7.97	8.49	8.24	0.10^{ns}	5.97	6.53	6.24	0.02*	$4.63e-16$****
MCF2L	9.49	10.21	9.87	$2.77e-4$***	8.29	8.34	8.31	0.60^{ns}	$1.02e-14$****
RHBDL3	5.84	6.92	6.41	$7.25e-4$***	4.63	4.69	4.66	0.99^{ns}	$4.32e-14$****
CBX7	9.49	10.20	9.86	$1.20e-5$****	8.85	8.86	8.85	0.85^{ns}	$4.43e-11$****
ABCA3	8.76	9.46	9.12	0.09^{ns}	7.40	7.46	7.43	0.67^{ns}	$2.87e-12$****
ZNF775	7.32	7.56	7.44	0.07^{ns}	6.65	6.79	6.72	0.21^{ns}	$4.10e-12$****

Table notes. Expression metrics for low and high expression of hsa-mir-1293 (with log2 normalized expression). μ_0: Average expression of class 0 (short-survival) in low / high miRNA expression, μ_1: Average expression of class 1 (long-survival) in low / high miRNA expression, μ_c: Average expression (both classes) for low / high miRNA expression, p_c: p-value of gene expression differences in low- and short-survival for low / high miRNA expression, p_T: p-value of gene expression differences (all data) for low / high miRNA expression. *Asterisks indicate statistical significance: (ns) no significative, (*) $p < 0.05$, (**) $p < 0.01$, (***) $p < 0.001$, (****) $p < 0.0001$.

These results support the hypothesis that miR-1293 plays a relevant regulatory role in HNSC. Moreover, the fact that higher gene expression, particularly under low miRNA expression, is associated with longer survival suggests a potential protective effect of these genes. However, some patients with good survival also show high miR-1293 expression and low gene expression, indicating that survival is not determined solely by these interactions but rather by a more complex set of biological and clinical factors.
