# Supplementary material for: RNACOREX - RNA coregulatory network explorer and classifier
Source: PLoS Comput Biol. 2025 Nov 3;21(11):e1013660. doi: 10.1371/journal.pcbi.1013660 (PMC12594346; doi:10.1371/journal.pcbi.1013660)
Supplement: S6 Text — Table A. Benchmarking.​ (PDF) [file pcbi.1013660.s006.pdf]

Benchmarking

Identifying disease-associated miRNA-mRNA interaction sets is usually approached in one of two ways: (i) using curated biological information, or (ii) applying general-purpose Bayesian network packages for network learning and inference. Comparing RNACOREX with the first type of approach is challenging, since methods based on curated information typically do not have the ability to make predictions on new datasets, and therefore the interactions cannot be validated outside the laboratory. Comparisons with generic Bayesian network packages are also limited, because these tools use all available data without considering the biological relevance of the interactions. Nevertheless, we conducted a comparison with one representative package. CGBayesNets is a Bayesian network software that implements Conditional Linear Gaussian classifiers (CLGs), the same model used in RNACOREX. Unlike RNACOREX, it does not incorporate curated information in the network learning process. However, it can still infer a network from raw data and make predictions on new datasets. Here, we present a small comparative analysis of RNACOREX and CGBayesNets, highlighting the strengths and limitations of both tools for post-transcriptional co-regulation network analysis.

- **CGBayesNets**

CGBayesNets [1] is a general-purpose package for learning and applying Conditional Linear Gaussian Bayesian Networks from input data, including several structure-learning and inference algorithms. It implements four structure-learning algorithms: K2, Pheno-Centric, Full-Exhaustive, and Naïve Bayes and two inference algorithms: Cowell and Variable Elimination. The package includes a basic pipeline, HybridBN, which uses K2 for structure learning and Cowell for inference. Users can modify parameters, select different algorithms, extract the network, and make predictions as needed. The package can be applied to genomics, metabolomics, and proteomics, learning a network from raw input data and performing inference on new data. However, the implemented algorithms are standard Bayesian network learning and inference methods and are not biologically informed.

- **Analysis**

Unlike CGBayesNets, RNACOREX incorporates curated information on the structure and characteristics of the interactions. To illustrate the importance of this prior information, we conducted a controlled experiment comparing RNACOREX and CGBayesNets. From each of the 13 TCGA databases used in the main experiments of the paper, 50 samples were randomly selected for training and 20 for testing, stratified by class. Both methods were applied using their default pipelines, and evaluated on both the training and independent test sets. Specifically in CGBayesNets, the `HybridBN` script was used for training the networks. The identified network was used for predicting on the test set using `PredictPheno`. In RNACOREX, networks from 2 to 200 interactions were compared using default configuration. The best performing network in the train set (both in accuracy and AUC) was selected and used for predicting over the test set.

Results are presented in Table A. Both CGBayesNets and RNACOREX showed excellent performance on the training data, reaching similar mean accuracies of approximately 93% and AUC-s of 0.98. However, their behavior differed notably when evaluated on the test sets. The accuracy and AUC obtained with RNACOREX were consistently higher than those achieved with CGBayesNets. Across the 13 analyzed datasets, RNACOREX achieved an average accuracy of 61% and AUC of 0.69, whereas CGBayesNets performed only slightly better than random assignment, with an average accuracy of 52% and AUC of 0.51.

To assess whether these performance differences were statistically significant, we conducted a significance analysis comparing the test set results from both methods. The Shapiro–Wilk test did not reject normality for any of the outputs, although the small number of observations ($n = 13$) limits the strength of this conclusion. For accuracy, the evidence was insufficient to confirm a consistent difference between methods, as p-values were slightly above 0.1 ($p = 0.1104$ for the paired t-test and $p = 0.1254$ for the Wilcoxon test). In contrast, the AUC differences were statistically significant, indicating that RNACOREX achieved superior performance on the test data ($p = 0.0012$ for the paired t-test and $p = 0.0034$ for the Wilcoxon test).

These results highlight the advantages of incorporating biological constraints into network inference. CGBayesNets relies solely on statistical associations, including all interactions that appear relevant,

Table A. Benchmarking.

	CGBayesNets			RNACOREX		
**Disease**	Acc	AUC	k	Acc	AUC	k
BRCA	0.94 / 0.65	1.00 / 0.61	24	0.98 / 0.60	0.99 / 0.70	133 / 131
COAD	0.88 / 0.55	0.93 / 0.54	16	0.92 / 0.90	0.99 / 0.91	94 / 175
HNSC	0.90 / 0.55	0.97 / 0.50	17	0.82 / 0.35	0.95 / 0.55	7 / 176
KIRC	0.96 / 0.55	1.00 / 0.49	22	0.90 / 0.75	0.96 / 0.81	48 / 69
LAML	0.96 / 0.50	1.00 / 0.56	37	0.96 / 0.80	0.99 / 0.89	147 / 147
LGG	0.94 / 0.60	1.00 / 0.60	32	0.90 / 0.50	0.97 / 0.70	190 / 143
LIHC	0.96 / 0.45	1.00 / 0.40	32	0.96 / 0.65	0.98 / 0.71	124 / 126
LUAD	0.90 / 0.40	0.95 / 0.35	14	0.98 / 0.55	1.00 / 0.68	61 / 60
LUSC	0.92 / 0.40	0.96 / 0.37	19	0.96 / 0.65	0.99 / 0.65	193 / 199
SARC	0.84 / 0.50	1.00 / 0.54	11	0.96 / 0.70	0.99 / 0.77	72 / 192
SKCM	0.96 / 0.50	0.96 / 0.66	29	0.94 / 0.50	0.99 / 0.55	70 / 98
STAD	0.98 / 0.60	1.00 / 0.51	35	0.96 / 0.40	0.99 / 0.47	140 / 167
UCEC	0.94 / 0.50	0.98 / 0.49	16	0.92 / 0.55	0.97 / 0.61	82 / 70

Table notes. Classification metrics for CGBayesNets and RNACOREX in the 13 databases. Accuracy and AUC are shown in (train / test) format. k represents the number of interactions of the selected network. In RNACOREX, k is defined as (best accuracy network / best AUC network) format.

regardless of biological plausibility. Without biological constraints, unrealistic interactions can appear, such as mRNA–miRNA links, if their expression patterns seem relevant. This makes the networks fit the input data very well but often leads to overfitting and poor generalization to new datasets. RNACOREX, by contrast, filters interactions according to biological and structural criteria, reducing the number of input variables and producing networks that are both biologically more meaningful and better able to generalize beyond the training data.

References

[1] McGeachie MJ, Chang H-H, Weiss ST. CGBayesNets: Conditional Gaussian Bayesian Network Learning and Inference with Mixed Discrete and Continuous Data *PLOS Computational Biology.* 2014 Jun, 10(6).
