# Supplementary material for: RNACOREX - RNA coregulatory network explorer and classifier
Source: PLoS Comput Biol. 2025 Nov 3;21(11):e1013660. doi: 10.1371/journal.pcbi.1013660 (PMC12594346; doi:10.1371/journal.pcbi.1013660)
Supplement: Attachment — Submitted filename: Review.pdf [file pcbi.1013660.s007.pdf]

Aitor Oviedo-Madrid
Institute of Data Science and Artificial Intelligence (DATAI), Universidad de Navarra
Ismael Sánchez Bella Building, Campus Universitario
31009 Pamplona, Navarra
Spain

Mark Alber, Ph.D.
Section Editor

PLOS Computational Biology

Dear Editor,

We, the authors, are pleased to present you our reviewed manuscript entitled

RNACOREX - RNA Coregulatory Network Explorer and Classifier

with manuscript number: **PCOMPBIOL-D-24-01999**.

We thank you for considering our manuscript and the careful handling of the review process. We provide a point-by-point responses to the Guest Editor and the three anonymous Reviewers.

We confirm that this manuscript is not under consideration by any other journal. The correspondence address is aoviedomadr@unav.es.

Yours sincerely,
Aitor Oviedo-Madrid, José González-Gomariz and Rubén Armañanzas.

RNACOREX - RNA Coregulatory Network Explorer and Classifier

Aitor Oviedo-Madrid, José González-Gomariz and Rubén Armañanzas

Point-by-point response to review comments

Dear Editor,

Here we present our responses to the anonymous reviewers' comments.

- Each question has been addressed as an independent point and appropriately numbered for reference.
- We also attach an amended manuscript with tracked changes in which deleted parts are crossed in ~~strikethrough~~ format, and additions are highlighted in blue color.
- The questions from the Reviewers are typed in *italic* format, whereas our responses are in plain format and **bolded** when a text cites manuscript excerpts.

We would like to thank the anonymous Reviewers for the time and effort in reviewing our work. We have incorporated all suggestions to the best of our ability, increasing the quality and readability of the manuscript. We consider that all concerns were solved in the amended version of the manuscript.

Comments from the Editor-in-Chief

Q1: We ask that a manuscript source file is provided at Revision. Please upload your manuscript file as a .doc, .docx, .rtf or .tex. If you are providing a .tex file, please upload it under the item type u2018LaTeX Source Fileu2019 and leave your .pdf version as the item type u2018Manuscriptu2019.

A1: Thank you for your guidance. We have uploaded both the manuscript source file (*Manuscript.tex*) and the PDF version (*Manuscript.pdf*) accordingly, selecting 'LaTeX Source File' for the .tex file and 'Manuscript' for the PDF as requested. We also include the supplementary material (*Supplementary.pdf*) and the response to reviewers (*Review.pdf*). The source code, data, and additional resources remain publicly available through the following platforms:

- GitHub: `https://github.com/digital-medicine-research-group-UNAV/RNACOREX`
- PyPI: `https://pypi.org/project/rnacorex/`
- Zenodo (Docker image with all dependencies): `https://zenodo.org/records/15805336`

Q2: Please provide an Author Summary. This should appear in your manuscript between the Abstract (if applicable) and the Introduction, and should be 150-200 words long. The aim should be to make your findings accessible to a wide audience that includes both scientists and non-scientists.

A2: Thank you for the clarification. We have included the required Author Summary in the revised version of the manuscript.

Q3: Please upload all main figures as separate Figure files in .tif or .eps format.

A3: We have uploaded all figures in .eps format labeled as FigX, for easy reference.

Q4: We have noticed that you have uploaded Supporting Information files, but you have not included a complete list of legends. Please add a full list of legends for your Supporting Information files (RNACOREX-main.zip) after the references list.

A4: We have included an index of all the figures contained in the Supporting Information in the main manuscript, placed after the references section. This allows the figures to be properly cited and referenced within the text. We appreciate your helpful comments that allowed us to improve the clarity of our submission.

Q5: Some material included in your submission may be copyrighted. According to PLOSu2019s copyright policy, authors who use figures or other material (e.g., graphics, clipart, maps) from another author or copyright holder must demonstrate or obtain permission to publish this material under the Creative Commons Attribution 4.0 International (CC BY 4.0) License used by PLOS journals. Please closely review the details of PLOSu2019s copyright requirements here: PLOS Licenses and Copyright. If you need to request permissions from a copyright holder, you may use PLOS's Copyright Content Permission form. Please respond directly to this email and provide any known details concerning your material's license terms and permissions required for reuse, even if you have not yet obtained copyright permissions or are unsure of your material's copyright compatibility. Once you have responded and addressed all other outstanding technical requirements, you may resubmit your manuscript within Editorial Manager. Potential Copyright Issues:

- *Figure 1. Please confirm whether you drew the images / clip-art within the figure panels by hand. If you did not draw the images, please provide (a) a link to the source of the images or icons and their license / terms of use; or (b) written permission from the copyright holder to publish the images or icons under our CC BY 4.0 license. Alternatively, you may replace the images with open source alternatives. See these open source resources you may use to replace images / clip-art: https://commons.wikimedia.org, https://openclipart.org/.*

- *The following file contains screenshots: Library_instructions.pdf in RNACOREX-main.zip. We are not permitted to publish these under our CC-BY 4.0 license, websites are usually intellectual property and are copyrighted. This includes peripheral graphics of the web browser such as icons and button. We ask that you please remove or replace it.*

A5: All figures included in the manuscript, supplementary material, and revision have been manually created by the authors. Additionally, the screenshots previously included in the GitHub repository (specifically in Library_instructions.pdf within RNACOREX-main.zip) have been removed, ensuring that no copyrighted images or materials remain. Therefore, no permissions are required for any figure or image used.

Q6: Please amend your detailed Financial Disclosure statement. This is published with the article. It must therefore be completed in full sentences and contain the exact wording you wish to be published.

- *State the initials, alongside each funding source, of each author to receive each grant. For example: "This work was supported by the National Institutes of Health (####### to AM; ####### to CJ) and the National Science Foundation (####### to AM)."*
- *State what role the funders took in the study. If the funders had no role in your study, please state: "The funders had no role in study design, data collection and analysis, decision to publish, or preparation of the manuscript."*
- *If any authors received a salary from any of your funders, please state which authors and which funders.*

A6: Thank you for your comments. We have included the initials of the authors receiving each grant alongside funding reference in the Financial Discolsure Statement, as recommended. We also include that funders had no role in study design, data collection and analysis, decision to publish, or preparation of the manuscript.

Reviewer 1

Q: The authors present RNACOREX, a computational method for analyzing miRNA-mRNA interactions using a combination of structural information from existing databases and functional information derived from expression data. The methodology culminates in the construction of a Conditional Linear Gaussian (CLG) classifier. The comments will be sorted by the supplementary material sections as look like an extended version of the main manuscript.

A: Dear Reviewer, thank you for your valuable comments and suggestions regarding our manuscript. We have carefully considered your remarks and provided detailed responses in the following pages.

Q1: The structural information score aims to minimize false interactions resulting from the Open World Assumption. One of its key components, the consistency index, evaluates whether a given interaction is reported in one, two, or all three databases. However, one of these databases exclusively contains experimentally validated miRNA–target interactions. The presence of an interaction in this database should be interpreted as biologically confirmed rather than merely inferred. How is this aspect incorporated into the scoring mechanism? Additionally, how many interactions present in miRTarBase have been discarded or assigned a low score by this method?

A1: Thank you for your comment regarding the weighting of validated versus predicted interactions in the calculation of the structural score. We appreciate the opportunity to clarify this point. The structural score is based on two components: the number of databases in which a given interaction appears and the consistency index, which measures the similarity between pairs of databases. This index serves to increase the weight of an interaction when the supporting databases returns similar interaction sets, even overlapping ones. It is true that, in the current algorithm, validated and predicted interactions are treated equally; no additional weight is assigned to an interaction simply because it has been experimentally validated. This choice was deliberate, as one of the objectives of the model is to remain capable of discovering potentially novel, yet unvalidated, interactions. Assigning an extra weight to validated interactions would likely bias the network composition towards only those interactions that have already been experimentally confirmed, reducing the exploratory power of the model.

Our analysis indicates that approximately 70% of the validated interactions present in miRTarBase are also found in DIANA and TargetScan. In contrast, only 5% and 12% of DIANA and TargetScan's predicted interactions, respectively, are shared between these two sources. This inherent asymmetry causes validated interactions to appear more consistently across multiple databases, thus benefiting from the consistency index and structural score without the need for manual reweighting. However, we acknowledge that having only one database of validated interactions (miRTarBase) versus two databases of predicted interactions (DIANA and TargetScan) could create an imbalance that might disadvantage validated interactions. To further balance the number of data sources, we have included TarBase in the package, an additional database of experimentally validated interactions. This database will increase the representation of validated data. Still, preliminary analyses show that overlap between validated databases remains limited (only about 5–7% of interactions are shared between them) reflecting possible inconsistencies among these datasets. This lack of overlap suggests that even experimentally validated data may not be fully reliable, providing further justification for not manually increasing their weight within the scoring system.

In summary, while the concern regarding the potential under-weighting of validated interactions is valid, the structure of our scoring approach and the characteristics of the databases themselves contribute to mitigating this issue. Furthermore, artificially increasing the score of validated interactions could reduce the discovery potential of the model and introduce unintended bias. To clarify this point we have included the following paragraph in the manuscript when presenting the structural information score:
"Since the package is intended to serve as a tool for discovering new potential, yet unvalidated, interactions, no distinction in weighting is made between validated and predicted interactions. Nevertheless, validated interactions tend to be more consistently represented across all databases compared to predicted ones, thus benefiting from the consistency index

and the structural score without the need for manual adjustment".

Q2: The equation (2) for the structural information score lacks clarity in how exactly the normalized consistency index contributes to the final score. The relationship between $I_{norm}(A, B)$ and w_{ij}^{ab} should be explicitly defined.

A2: We have revised the manuscript to clarify how this score is computed, so that it can be properly understood. In essence, the structural information score aims to capture the curated knowledge available regarding a given interaction. The formula $SI_{ij} = \sum_{k=1}^{4} \sum_{l=1}^{4} I_{norm}(S_k, S_l) * w_{ij}^{kl}$ is based on two components: the number of sources (engines, databases) in which an interaction appears, and the consistency index across the engines where it is present. Specifically, w_{ij}^{kl} indicates whether a (i, j) interaction is present in an (S_k, S_l) pair of engines (binary), while the normalized consistency index I_{norm} represents the agreement level between interactions from each engine pair. The score value is calculated by iterating over all pair of engines as the sum of the consistency indices across all engine pairs where the interaction is found. The "normalization" term simply refers to the raw consistency indexes (as obtained from the formula of the consistency index) are normalized so that their total sum equals 1. This means that if an interaction appears in all engines, it will receive a structural score of 1.
We have included the following paragraph along with the mathematical definition of the structural information score in the main text:
"The structural score SI is based on two components: the number of databases in which the interaction is present and the consistency index among the pairs of databases where it appears. The consistency index measures the similarity between two sets of interactions, assigning greater weight to an interaction if it occurs in databases that are themselves similar. The score is computed by summing the consistency indices of all pairs of databases in which the interaction is present. This index is normalized such that the total sum of indices across all database pairs equals 1, ensuring that the structural score of interactions present in all databases is equal to 1."

Q3: Also, RNACOREX seems to consider only the already existent pairs from other databases, excluding the discovery of newly interactions. The authors should clarify whether the filtering process might introduce biases by potentially excluding novel but biologically relevant interactions not yet documented in databases.

A3: As the Reviewer correctly points out, the RNACOREX approach introduces a bias in favor of interactions that have already been predicted or validated. As a result, the resulting coregulatory networks are composed only of those known or predicted interactions, overlooking entirely novel ones. While this approach does limit the discovery of completely new interactions, it is important to note that many of the interactions included via TargetScan or DIANA are still only computational predictions and have not been experimentally validated. Therefore, identifying some of these interactions across different scenarios could serve as a valuable first step toward their future biological validation. This approach was deliberately chosen to reduce the number of false positives typically generated by computational approaches driven exclusively by expression levels. Of course, removing this prior filtering would increase the chances of detecting completely novel interactions, but at the cost of also including a large number of spurious ones. Given the high cost of experimental validation, we prioritized specificity over sensitivity, aiming to ensure a higher level of consensus and confidence in the identified interactions.

In Section 2 of the manuscript, we have included a new subsection (2.2. Filtering) including the next paragraph:
"RNACOREX leverages these databases to perform an initial biological plausibility filter. Specifically, it retains only those interactions that are supported by at least one of the previously mentioned sources (either as a prediction or as experimental evidence) while discarding interactions absent from all of them. The reasoning behind this is that if an interaction has not even been predicted using well-established features such as sequence complementarity, it is unlikely to occur in a real biological context. This deliberate filtering reduces the likelihood of including spurious interactions, though at the cost of overlooking entirely novel interactions.

However, as many entries from TargetScan and DIANA remain computational predictions without experimental validation, recovering these interactions in independent scenarios could guide future biological validation."

Q4: While kernel density estimation is a reasonable approach, the authors should address how they handle the curse of dimensionality, particularly for datasets with limited samples. The default of 10 trapezoids for numerical integration seems arbitrary. The authors should provide justification for this choice or demonstrate its adequacy through validation experiments.

A4: We thank the reviewer for the insightful comment. We acknowledge that the curse of dimensionality could be a problem when developing kernel density estimation with limited samples. However, in our work, KDE is applied only to 1D or 2D distributions (e.g., variables X and/or Y), where the curse of dimensionality is not a significant issue. In such low-dimensional settings, kernel density estimation is known to perform reliably. Therefore, we believe KDE remains a valid and robust approach in our case.

Regarding the trapezoid number selection, it is correctly pointed out that the decision to select 10 trapezoids as the default value is to some extent arbitrary. To address this, we have conducted a more in-depth analysis of both computational performance and estimation accuracy in order to justify the default choice. The results of this analysis are presented in the Supplementary Material Section 3.2 'Precision Setting'. Based on these findings, we have revised the default number of trapezoids to 20.

Q5: The alternating selection approach from structural and functional rankings is interesting but lacks theoretical justification. The authors should explain why this approach is preferable to other ranking combination methods (e.g., weighted combinations). The method for handling ties in either ranking is not specified but could significantly impact results.

A5: It has been observed that methodologies relying on a single computational approach for interaction discovery, whether based on machine learning or structural information, tend to identify a large number of spurious interactions. One of the main strengths of RNACOREX lies in the combination of those two approaches, incorporating both curated and empirical information through expression analysis. Therefore, the decision to use the alternative approach was made consciously, as it allows for approximately 50% of the elements to be derived from each of these two complementary sources.

However, to offer users a broader set of options, we have decided to expand this aspect by allowing the selection of different methodologies for constructing the interaction ranking. In addition to the *alternative* approach, we now include the *structural*, *functional*, and *weighted* modes. The *structural* and *functional* approaches build the ranking using only one specific type of information. In contrast, the *weighted* approach assigns a weight to each score, so that the combined score determines the inclusion order of interactions, prioritizing those with higher aggregated values. In our experiments, we have chosen to apply the *alternative* approach as the default strategy, since it aligns with the intended design philosophy of the package: giving equal importance to both sources of information. However, users can freely explore and customize the other methods according to their specific research needs or hypotheses.

Regarding the procedure for resolving ties, we acknowledge that was not explicitly described in the main text, and we apologize for the omission. This clarification is particularly important in the case of the structural score, where ties are frequent. It is important to note that, by the time ties are resolved, all remaining interactions have already passed a filtering process and are considered to have sufficient supporting evidence to be included in the analysis. Therefore, tie-breaking decisions are applied only among interactions that are already biologically meaningful. Among this interactions, certain elements can exhibit a very strong association with the class of interest, often resulting in the monopolization of a large number of interactions. To mitigate this effect and promote the discovery of a broader set of regulatory pathways, a tie-breaking strategy based on node connectivity is applied. Specifically, when two or more interactions receive the same score, preference is given to those involving nodes that are less connected to the rest of the network. This encourages the inclusion of interactions that are potentially more isolated and novel, rather than repeatedly

selecting highly connected nodes. For this purpose, after filtering, the combined degree of each interaction is calculated as the sum of the degrees of both nodes involved. The degree of a node is defined as the total number of its incoming and outgoing connections, considering all interactions within the full set of potential interactions identified after filtering. Interactions with lower combined degree values are prioritized in the ranking, ensuring that ties are resolved by favoring interactions linked to less integrated elements within the network.

We included information about these new methods in the 2.4 'Interaction Hierarchy' and 2.6 'Software Features' sections of the manuscript. An extended description of the methods for tie-breaking strategy is also available in the 3.3. Section of Supplementary Material.

Q6: Figure 2 illustrates the post-transcriptional network generated. Two main types of interactions can be identified:

1. *Those with higher Structural Information than Functional Information.*
2. *Those with the inverse pattern.*

Is there a systematic bias favoring structural or functional information during training? If so, how does this affect prediction accuracy? It might also be useful to check feature importance scores from the classifier to support this claim.

A6: Thank you for this comment. As stated in the manuscript and in the previous question (Q5), the alternative approach deliberately integrates both structural and functional information by selecting 50% of the top interactions ranked by structural scores and 50% by functional scores. This ensures that both data sources contribute equally to the final network, without favoring one type of information during selection. As a consequence, some interactions included in the network may appear "unbalanced", *i.e.*, they may display a high score in one dimension (structural or functional) while having a relatively lower score in the other. This outcome is expected and intentional, as the approach is designed to capture the most relevant interactions according to each type of information, regardless of their score in the complementary dimension.

Therefore, this approach does not introduce a systematic bias toward either structural or functional information but rather enforces equal representation from both sources. The purpose is to provide users with a comprehensive network that reflects both aspects equally. As mentioned in A5, we have now included other ranking strategies in the package, allowing users to explore different weighting schemes depending on their specific research questions or preferences.

Regarding the suggestion to examine feature importance scores, we would like to clarify that the structural and functional information used in RNACOREX already serve as measures of association between an interaction and the class label. Specifically, the functional information score is a quantitative metric derived from conditional mutual information, capturing how strongly an interaction (*i.e.*, a miRNA–mRNA pair) is associated with the class variable. These values are directly available after training through the `.connections_` attribute of the model. In addition, the conditional Gaussian distributions implemented in the underlying Bayesian network provide further insight into dependencies between variables. The coefficients stored in `.clgc_['beta']` and `.clgc_['sigma']` reflect how each node is influenced by its parents. These can be interpreted as local feature importance indicators, quantifying the strength of the statistical relationship between a node and its direct causes in the network.

While global feature importance measures are more difficult to derive in this probabilistic setting and are not currently implemented in the RNACOREX package, we agree that such functionality would be valuable. We have included this point in the Availability and Future Directions section:
"In addition, incorporating methods to estimate global feature importance-for example, metrics that quantify the overall relevance of individual RNAs or interactions across the network-could offer deeper insights into the key drivers of classification and improve the interpretability

of the models.”

Q7: A Docker container with all necessary data and a well-organized folder structure would significantly enhance usability, enabling users to seamlessly run the methods and better understand the required inputs.

A7: Thank you for the suggestion, we agree that providing a Docker container improves usability. We have uploaded the complete package to Zenodo, including all necessary data and a well-organized folder structure. The container is fully self-contained and can be launched automatically without requiring any additional dependencies. It is available under the ‘DOI: 10.5281/zenodo.15805336.‘

Q8: The differential expression analysis (DEA) between Short and Long Survival groups is used to reduce the number of mRNA candidates before constructing the network. This is a reasonable approach to remove non-variable mRNAs that are unlikely to influence overall survival. However, filtering by p-value instead of FDR is statistically incorrect when applying thousands of tests. Moreover, the justification for further filtering—“genes are filtered by RNACOREX using databases of predicted and validated interactions”—raises concerns about the DEA approach. If RNACOREX is already filtering mRNAs based on documented interactions, why apply a questionable pre-filter beforehand?

A8: Thank you very much for your comments. You are absolutely right in pointing out that not applying FDR correction is inappropriate when conducting multiple simultaneous tests. In the revised version of the experiments, we have addressed this issue by applying the correction as recommended. We include a brief explanation of the preprocessing in the manuscript and full description is included in the Supplementary Material.

Regarding your question about the need to perform this DEA analysis, it is important to note that the filtering performed by RNACOREX during the induction of the network is based solely on whether interactions have been predicted or experimentally validated at some point, but it does not consider the phenotype under study. Therefore, in designing the experiments, we considered it relevant to apply an initial filtering step based on phenotype. This step ensures that only mRNAs that are differentially expressed in the specific problem under investigation are selected. In this way, we remove interactions that may be structurally relevant but have no connection to the phenotype of interest. These differentially expressed elements will subsequently be filtered by RNACOREX if they are not part of any interaction identified by the MicroRNA–Target Interaction (MTI) engines. Anyway, the DEA prefiltering is not a direct part of the library and can be skipped.

Q9: Supplementary Figure 3 is not properly referenced and contains a typo (“enaugh” → “enough”).

A9: Thank you for pointing this out. We have corrected the typo in Supplementary Figure 3. We have also fixed the reference to ensure the figure is properly cited in the text.

Q10: While Random Forest (RF), Gradient Boosting (GB), and Support Vector Machine (SVM) are widely used in vector-based classification, they are not inherently designed for graph-based classification. To establish a baseline for evaluating RNACOREX, I suggest comparing it against more graph-specific state-of-the-art methods, such as:

- *Graph Neural Networks (GNNs)*
- *Graph Kernels (e.g., Weisfeiler-Lehman kernel + SVM).*

A10: We thank the reviewer for this valuable suggestion. We fully agree that graph-specific methods offer a more appropriate baseline for evaluating RNACOREX. In response, we have implemented both a Graph Neural Network (GNN) and a Graph Kernel approach based on the Weisfeiler-Lehman kernel combined with an SVM classifier, as recommended. We kept the comparisons with the original vector-based models (Random Forest, Gradient Boosting, and SVM) to provide a broader context for performance evaluation. The results of all comparisons, including the newly added graph-based models, are now available in Table 1 and Figure 3 in the 3.3 Section of the revised manuscript. Specific implementation details for both the GNN

and the graph kernel models are included in the Supplementary Material.

Q11: The section begins by claiming a 10%–30% accuracy increase, but this should be clearly visualized in a single figure.

A11: Thank you for the suggestion. In the revised version, we have added a new Figure 3, which clearly illustrates the accuracy of RNACOREX in comparison with the other methods, as well as against a random assignment (50%). This visualization helps better contextualize the reported performance values.

Q12: Additionally, does accuracy correlate with the number of mRNAs used? At first glance, datasets with the highest performance (LGG and UCEC, with 4,729 and 3,000 mRNAs, respectively) contain substantially more mRNAs than the lowest-performing datasets (SKMC and LUSC, with 1,270 and 1,079 mRNAs). Is there a statistical correlation (spearman) between accuracy and mRNA count?

A12: Thank you for the insightful observation. Indeed, it is reasonable to expect that using a larger number of input mRNAs, resulting in more potential connections, could lead to more comprehensive networks and, consequently, improved performance metrics. After re-running the experiments and implementing the new DGE pipeline, we specifically investigated the relationship between the number of mRNAs (and connections) and the predictive accuracy. The results of this investigation are shown in the following Figure R1.

The results show no significant correlation. Specifically, we observed an almost null trend when comparing accuracy to the number of connections. These analyses were conducted using both the mean accuracy across all tested values of k and the maximum accuracy achieved at the optimal point k. Therefore, we conclude that neither the number of genes nor the number of potential interactions appears to significantly influence the predictive accuracy of the models.

Q13: Other figure-related concerns: Figures for different sample results (Supp. Fig. 4–15) lack proper descriptions.

- *What is the y-axis?*
- *What does the blue ribbon represent?*
- *What do the rank and metrics indicate?*

Since "Test" remains constant across three figures, and the different models are color-coded, it would be more readable if all lines were presented within a single graph.

A13: We thank the reviewer for the helpful observation. We agree that the original figures lacked some clarity. As a result, we have decided to remove those figures and revise the way we present those results. Specifically, we now compare the models using a simplified boxplot that shows the distribution of accuracy for each method. The new figure is now included in the main manuscript (please see Figure 3). Additionally, we provide two new summary tables: one in the main manuscript summarizing the overall performance metrics of RNACOREX, and another in the Supplementary Material presenting the complete performance metrics for all models evaluated.

Q14: The final validation of the tool is underdeveloped. Given that miRNA–mRNA interactions are cancer-specific and focus on overall survival shifts, I would expect an analysis demonstrating that the mRNAs in predicted miRNA–mRNA pairs are linked to biologically relevant pathways. I strongly suggest performing such an enrichment/pathway analysis to better showcase the tool's potential.

A14: We appreciate the reviewer's comment. The primary focus of this manuscript is the presentation of the RNACOREX software, including the underlying methodology and the technical capabilities of the tool. As such, we have prioritized demonstrating its performance and utility rather than conducting an in-depth biological analysis of the predicted miRNA–mRNA interactions.

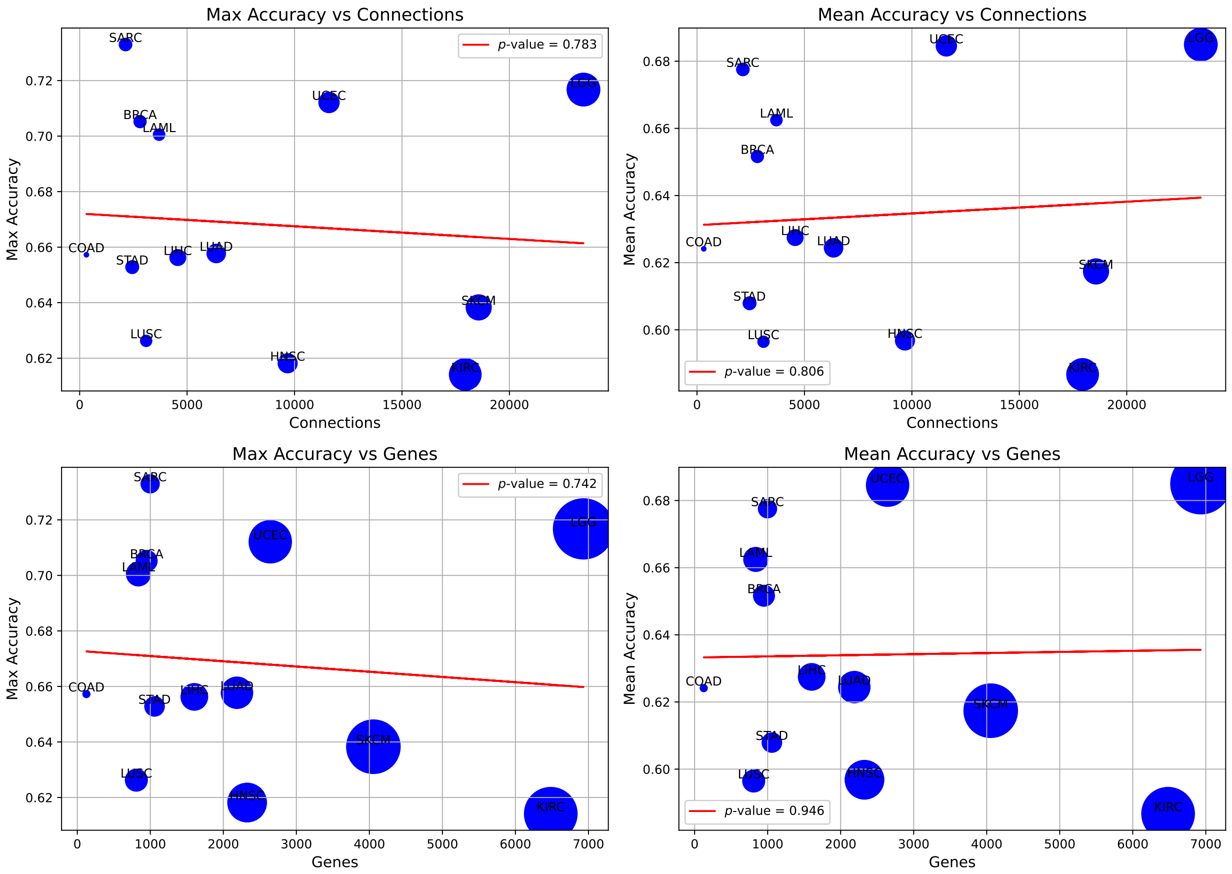

Figure R1: **(A12) Relation between accuracy and input elements**. This Figure represent the existing correlation between the number of input elements and the accuracy of the networks. For comparison, the accuracy of the network with the best performance and the mean accuracy between all k values is used. This values are compared to the number of differentially expressed mRNAs in each database and the number of potential interactions after filtering with the engines. The size of the bubbles represent number of genes for connection plots and number of connections for gene plots.

The results presented are intended to be illustrative, showing the kinds of outputs RNACOREX can generate. We included a preliminary analysis highlighting the most commonly inferred elements and interactions, along with references from the literature linking them to various cancers. However, we agree that a more comprehensive biological interpretation through pathway or enrichment analysis would be a valuable next step.

That said, such analyses are currently beyond the scope of the tool's core functionalities and the length constraints of Software articles in PLoS Comp Biol. While we did not incorporate enrichment or pathway analysis in this work, we have now included this perspective in the revised Availability and Future Directions section of the manuscript, where we outline potential extensions and applications of the tool, including its integration with pathway-level analysis frameworks.

Q15: Supplementary Figures 17–29 add little to no value, as they are neither analyzed nor referenced.

A15: We have removed these figures from the Supplementary Material. For illustrative purposes, we have retained one representative network corresponding to the BRCA cancer type, which is now included in the main manuscript.

Q16: miRTarBase was updated on January 6, 2025—newly added interactions could be used to validate the predictions.

A16: Thank you very much. You are correct in noting that miRTarBase was updated to its new version 10.0 in early 2025. With this update, the database has increased from approximately 2.2 million miRNA-mRNA validated interactions to nearly 4 million. This latest version is now incorporated into the package, including a larger number of validated interactions.

Q17: Why not leverage TCGA's paired tumor–healthy data? TCGA provides a well-characterized, paired dataset, facilitating a clearer comparison between tumor and normal samples. Why did the authors opt for a tumor-specific survival drift approach rather than identifying miRNA–mRNA interactions that directly drive the oncological phenotype? Would integrating paired tumor-normal data improve the model's interpretability?

A17: We thank the reviewer for the comment. TCGA samples include a barcode that identifies the sample type: tumor (00–09), peritumoral (10–19), or normal/control (20–29). Although we initially considered using control samples (from healthy individuals), such samples are generally unavailable in TCGA. One alternative could be comparing peritumoral and tumor samples, but the number of available cases is also very limited. To include healthy samples, it would be necessary to complement TCGA data with external datasets such as GTEx. This option was considered; however, the number of usable samples with matched miRNA-mRNA expression data was lower than when using only TCGA tumoral data. Given this, we decided to focus on tumor samples, which allowed for a more consistent analysis. Nevertheless, we agree that integrating healthy samples would be an interesting direction for future work.

Q18: The abstract highlights RNACOREX's "inherent explanatory capability related to the pathology being studied", yet no supporting results are provided. No bibliographical validation is offered to demonstrate that the predicted miRNA–mRNA pairs correlate with changes in overall survival across different cancers.

A18: As stated in the manuscript, when referring to RNACOREX's "inherent explanatory capability", we highlight a general and fundamental property of Bayesian network models, such as the CLG structure employed here. This type of model explicitly represents the probabilistic dependencies (between miRNAs and mRNAs in this case) through a graphical structure (the network) and associated conditional probability parameters. These elements provide insight into which molecular interactions (pairs of elements) contribute to the classification outcome, thus offering an explanation for the model's predictions beyond mere input–output mapping.

While RNACOREX generates these interpretable networks for each cancer dataset analyzed, the current

study focuses on the methodological development and validation of the tool itself, and therefore does not include an exhaustive biological analysis of all predicted interactions. We do however present a brief discussion highlighting the most frequently selected molecular elements and interactions across different cancer types, which may suggest potential pan-cancer regulatory roles. For these elements, we provide supporting bibliographical references documenting their previously reported involvement in cancer-related processes. This evidence offers preliminary biological validation and indicates the potential of RNACOREX to guide the formulation of new hypotheses in cancer research. A more comprehensive biological interpretation of the inferred networks is intended for future work.

Reviewer 2

Q: RNACOREX was developed for the classification capability for cancer survival based on miRNA-mRNA interactions and their functional relevance within a Conditional Linear Gaussian (CLG) Bayesian network framework. However, some aspects of the methodology and presentation require clarification and more detailed justification before publication. Key details regarding the implementation of the structural and functional information scoring, the network learning process within the CLG framework, and a more in-depth discussion of the biological interpretation of the results and network structures are needed. The performance evaluation could also benefit from additional context and possibly alternative metrics.

A: Thank you very much for your constructive comment. We have addressed each of your points to the best of our ability and have made the necessary changes to the manuscript to improve the clarity, justification, and overall presentation of the methodology, results, and biological interpretation.

Q1: The manuscript would benefit significantly from a more detailed, practical case study. Currently, the authors highlight a few pan-cancer miRNA biomarkers. However, deeper biological insight into specific miRNA-mRNA interactions is lacking. I suggest the inclusion of a case study focused on a single cancer type, analyzing how the top-ranked interactions relate to known biological pathways or potentially novel mechanisms. A deeper biological analysis of the network structures and the roles of the highlighted interactions would significantly strengthen the manuscript's impact.

A1: We appreciate the suggestion and fully agree that a deeper biological analysis focused on a single cancer type could offer important insights and significantly strengthen the manuscript's impact. While the main objective of this work is to introduce and describe the RNACOREX package, we have made an effort to assess the biological relevance of the top-ranked elements identified by the tool by verifying whether these interactions and molecules have been previously associated with cancer in the literature. However, since the aim of this manuscript is not to conduct an exhaustive biological case study, but rather to present the design, implementation, and general applicability of the software, we opted not to include more detailed analyses such as pathway enrichment or tissue-specific interpretation at this stage. This is also in agreement with the constraints of the Software articles in PLoS Comp Biol, which this manuscript is intended for.

That said, we fully agree that applying RNACOREX to a specific cancer type and conducting a more in-depth biological investigation—exploring the functional roles of top-ranked interactions and their involvement in known or novel pathways—would be a valuable next step. We have included this perspective in the 'Availability and Future Directions' section of the manuscript:
"A logical next step in the development and application of RNACOREX would be to focus on specific tissues or conditions, performing a more in-depth biological analysis of the identified interactions. This would include a systematic functional interpretation, such as pathway and enrichment analyses, to better understand the biological roles of the selected networks in a given context. Incorporating this level of analysis would greatly enhance the biological relevance and interpretability of the results, and could be considered for integration into the pipeline in future versions of the package."

Q2: The manuscript would benefit from specifying the advantages of MTI interaction in survival prediction over simply a group of miRNAs or a group of mRNAs.

A2: Thank you for your comment. We agree that the manuscript did not clearly highlight the specific advantage of using miRNA–target interactions (MTIs) for survival prediction. Several studies have shown that including not only the individual expression levels of molecular elements but also the relationships between them through miRNA–target interactions (MTIs) improves the predictive performance of the models. Moreover, using MTIs instead of analyzing elements individually allows for the identification of more complex regulatory relationships possibly underlying pathological phenomena. This broader understanding enables us to determine not only which elements are involved in disease development, but also how they interact with one another. To explain this point, we included the following sentence in the introduction of the manuscript:

“BNs not only leverage raw expression levels but also allow the incorporation of prior knowledge on miRNA–target interactions (MTIs) into the model structure, enhancing biological interpretability and improving predictive performance.”

We based these comments in the next references: “Gade S, et al. Graph based fusion of miRNA and mRNA expression data improves clinical outcome prediction in prostate cancer. *BMC Bioinformatics.* 2011, Vol. 12, 488.” and “Xu J, et al. Unveiling Novel miRNA-mRNA Interactions and Their Prognostic Roles in Triple-Negative Breast Cancer: Insights into miR-210, miR-183, miR-21, and miR-181b. *Int J Mol Sci.* Feb 2025, 26(5).”

Q3: The performance or novelty of RNACOREX should be compared with previous methods such as CG-BayesNets; miRNA–Target Gene Regulatory Networks: A Bayesian Integrative Approach to Biomarker Selection with Application to Kidney Cancer; A Bayesian framework that integrates multi-omics data and gene networks predicts risk genes from schizophrenia GWAS data and so on.

A3: Thank you for this observation. While there are various tools for either MTI discovery or Bayesian network modeling, few integrate both aspects in a unified pipeline, as RNACOREX does. Most MTI-focused tools are limited to identifying interactions or constructing networks without incorporating them into predictive models (i.e. “Chekuouo T, Stingo FC, Doecke JD, Do K-A. miRNA–Target Gene Regulatory Networks: A Bayesian Integrative Approach to Biomarker Selection with Application to Kidney Cancer *Biometrics.* 2015 Jun, 71(2), pp. 428-438.” and “Wang Q, et.al. A Bayesian framework that integrates multi-omics data and gene networks predicts risk genes from schizophrenia GWAS data *Nat Neurosci.* 2019 May, 22(5), pp. 691-699.”). Conversely, existing Bayesian network tools, such as CGBayesNets, lack dedicated modules for MTI discovery and rely solely on data-driven learning, which may introduce biologically implausible interactions. Moreover, CGBayesNets is not actively maintained and has not been applied to miRNA-mRNA data, limiting comparability. This differences in objectives and scope, make direct performance comparison with RNACOREX unfeasible. We have briefly commented these issues in the last paragraph of the introduction:
“The novelty of RNACOREX lies in its integrated pipeline, which provides a comprehensive framework specifically adapted to the field of transcriptomics. It enables researchers to easily identify relevant sets of interactions and use them to construct predictive models. While there are existing methods for identifying interaction sets, these typically do not incorporate curated biological information or are not designed to build predictive models. Similarly, although Bayesian network approaches capable of handling conditional linear Gaussian (CLG) models do exist, they are not adapted to work with miRNA–mRNA interactions (MTIs), limiting their applicability in this domain.”

Q4: The use of the Conditional Linear Gaussian (CLG) classifier is briefly justified by stating that it is suitable for gene expression data. A clearer theoretical rationale for its choice—possibly compared to alternative probabilistic models—would improve the manuscript’s methodological transparency.

A4: Thank you for this observation. In transcriptomic data analysis, Bayesian networks are often avoided due to the need to discretize continuous variables, which can reduce information and model performance. RNACOREX, however, works with continuous variables (miRNA and mRNA expression levels) and a discrete target variable (the class label). The CLG model is especially appropriate in this context, as it enables the construction of Bayesian networks with both continuous and discrete variables without having to discretize the continuous ones. Additionally, using Gaussian distributions enables more efficient inference, making CLG a practical and appropriate choice for our model.

We have revised the Conditional Linear Gaussian section, including a more clear explanation of the suitability of CLGs for the RNACOREX package:
“The networks inferred by RNACOREX consist of two types of variables: continuous expression variables (miRNAs and mRNAs) and a binary class variable (phenotype). This structure makes them ideally suited for representation using a Conditional Linear Gaussian (CLG) model, as it allows working directly with continuous data without the need for discretization,

and enables highly efficient inference by leveraging the Gaussian properties of the variables." We also include a bref comment in the introduction: **"With these interactions, RNACOREX implements CLG models- a specific kind of BN- to build Bayesian network classifiers through a graphical representation of the post-transcriptional regulatory network. These models are particularly well-suited to this problem, as they avoid the need for data discretization and take advantage of the properties of Gaussian distributions to enable more efficient inference.**

Q5: Please clarify the process of learning the CLG Bayesian network structure. Is a structure learning algorithm employed, and if so, which one and how does the interaction ranking influence it? How are the parameters of the CLG learned from the data?

A5: The network topology is not defined using a specific structural learning algorithm. Instead, a custom method has been developed, based on both structural and functional scores calculated for each interaction. In this approach, interactions (edges) are sequentially incorporated into the network according to their score values. These scores fully determine the inclusion order of the interactions in the network structure, defining an interaction hierarchy. RNACOREX does not aim to find an optimal or fixed number of interactions; rather, it is the user who defines how many interactions the final network should contain. If the user specifies a number k, the resulting network will consist of the top k interactions in the interaction ranking. Alternatively, if the user prefers RNACOREX to select the most appropriate network, they can define a maximum number of interactions and the package will choose the network within that range that yields the best classification performance. Regardless of the selected strategy, the structure of the resulting network is always defined by the ordering of the interactions according to their structural and functional scores. For instance, if the best-performing network includes 48 edges, those will be the first 48 interactions in the interaction ranking. We included the next brief explanation in the main manuscript:
"In RNACOREX, the topology of the network is fully defined by the interaction ranking and the number of interactions (k) set by the user. The network will be composed of the first k interactions in the interaction ranking."

Once the network topology is defined, node parameters are learned from the data. RNACOREX networks include discrete nodes (the class) and continuous nodes (mRNAs and miRNAs). In the CLG framework, continuous nodes follow Gaussian distributions conditioned on their parent nodes. There are two main cases:

1. Continuous nodes with only the class as a parent: These are modeled with one Gaussian per class, *i.e.*, $P(X_i|C) \sim N(\mu(C), \sigma^2(C))$, with parameters estimated from the data corresponding to each class.

2. Continuous nodes with the class and other continuous parents: These are modeled via class-conditional linear regression. For each class, the conditional distribution is Gaussian, with parameters estimated from a linear regression on the continuous parents: $P(X_i|\mathbf{Y}) = P(X|\mathbf{Y}, C) \sim N(\beta_0(C) + \beta^T(C) \cdot \mathbf{Y}, \sigma^2(C))$. The parameters of the linear regression can be easily estimated using the mean vector and covariance matrix of the node and its parents.

This approach allows for class-specific parameter estimation while preserving the dependency structure defined by the network topology. We have updated the Conditional Linear Gaussian Classifier part of the manuscript to make this explanation more accessible and rigorous (see Section 2.5 in main manuscript and Section 2.4 in Supp. Material). The specific calculations of β and σ parameters are presented in the Supplementary Material.

Q6: Related to Q4-5, key methodological details—such as data preprocessing steps (Supplementary Material Section 4.2 is referenced for preprocessing, but a brief mention in the main text would improve clarity), methods for dividing patients based on survival time (long/short) (how this cutoff was determined). A brief overview of these steps should be included in the main text for clarity and transparency.

A6: Thank you for your valuable suggestion. We have briefly included key methodological details in the main text to improve clarity and transparency, as you recommended. Specifically, we now provide a concise description of the data preprocessing steps and the method used to divide patients based on survival time,

including the criteria for determining the cutoff between long and short survival groups. Further details are also available in Supplementary Material Section 4.2 as referenced.

Q7: While the manuscript describes the general idea of structural and functional information scores and an interaction hierarchy, the specific mathematical formulations and weighting schemes are referenced to the Supplementary Material. These are fundamental to the RNACOREX methodology and should be clearly summarized in the main manuscript or provided with sufficient detail in the supplementary material (once accessible) to allow for critical evaluation and reproduction.

A7: Thank you for this important comment. We agree that the structural and functional scores are key components of the RNACOREX methodology. In response to your suggestion, we have now included the mathematical definitions of both scores directly in the main manuscript to ensure clarity (see Section 2.3 in main manuscript).

Q8: "The performance of the network with the best metrics computed by RNACOREX was compared to three state-of-the-art classification models: Random Forest, Gradient Boosting, and SVM." Please provide proper reference or rationale that these are considered state-of-the-art for this problem type. In addition, Table 1 only shows RNACOREX's performance for a subset of the datasets. A comprehensive comparison, presenting the key performance metrics for the mentioned models across the selected datasets, is necessary to allow readers to assess the relative performance of RNACOREX. A comprehensive figure would be much stronger to support your claims. It would also strengthen the manuscript to include the full performance comparison in the main text, rather than relegating it to supplementary materials.

A8: Thank you for the insightful comment. We agree that while Random Forest, Gradient Boosting, and Support Vector Machines are widely used and often considered strong baselines for classification tasks, they are not specifically tailored for graph-based problems. To address this, we have expanded our comparative analysis to include two additional methods that are better suited for graph-structured data: a Graph Neural Network (GNN) and a Graph Kernel approach (Weisfeiler-Lehman kernel combined with SVM). This allows for a more appropriate and meaningful evaluation of RNACOREX within its methodological context.

Regarding the presentation of results, we acknowledge that Table 1 previously provided only a partial view. We have now revised the manuscript to include RNACOREX's performance across all datasets and added a new figure (Figure 3) that comprehensively compares its performance with all five competing models. Detailed performance metrics for each model and dataset is also presented in the Supplementary Material to ensure full transparency and reproducibility.

Q9: While the supplementary files are linked at the end of the manuscript, they are either missing from the GitHub repository or difficult to locate there. The wording in the manuscript gives the impression that the files are available on GitHub, which may cause readers to overlook the fact that they are actually included at the end of the manuscript.

A9: Thank you for bringing this to our attention. You are absolutely right that the current wording in the manuscript could create confusion regarding the location of the supplementary materials. We have clarified this in the revised version. The supplementary materials, including all additional tables and figures, are provided at the end of the manuscript in the dedicated Supplementary Material document. The GitHub repository, on the other hand, hosts the source code for the RNACOREX library, the required information for running it, and a QuickStart example to facilitate its use. We have updated the manuscript to reflect this distinction more clearly.

Q10: Figure 1 provides a workflow, which is helpful. Improving the structure and clarifying the meaning of figures would benefit the manuscript.

A10: Thank you for your valuable feedback. The original version of Figure 1 may have been too complex, potentially making it difficult to interpret for readers unfamiliar with the methodology. In response, we have

revised and simplified the figure to focus more clearly on the key steps and components of the RNACOREX workflow. The updated figure aims to provide a more intuitive and accessible overview of the package's core functionalities.

Q11: While the manuscript is generally well written, there are several imprecise sentences. I recommend a thorough proofreading and language polishing to improve fluency and clarity.

A11: Thank you for your comment. We have carefully revised the manuscript to address this concern. In this updated version, we have made a concerted effort to improve the overall fluency and clarity of the language throughout the text.

Q12: Terms like "post-transcriptional network," "coregulatory network," and "regulatory network" are used interchangeably. Consider being consistent in definitions throughout.

A12: Thank you for this observation. We have revised the manuscript to address this issue. Specifically, we now use the term post-transcriptional network to refer exclusively to networks composed of miRNA–mRNA interactions. This is a specific type of coregulation network. When discussing about coregulatory networks, we are referring to sets of interactions that are associated with a specific pathological conditions. We have avoided the term 'regulatory network', as we consider 'coregulatory' to be more appropriate given the multiple elements involved. However, we retain the use of regulation to describe direct interactions between elements, such as in the expression **"the package assumes a regulatory flow from miRNAs to mRNAs."**

Q13: For readers less familiar with classification metrics, consider briefly explaining key metrics such as AUC, sensitivity, and specificity in the manuscript.

A13: Thank you for the suggestion. We fully agree that clarity is important, especially regarding key performance metrics. However, we believe that providing detailed explanations of standard classification metrics such as AUC, sensitivity, and specificity falls outside the scope of the manuscript, as these metrics are well-established and familiar to the target readership of the journal. Moreover, formal definitions of these metrics are readily available in standard literature. Nevertheless, we have ensured that these terms are used clearly and consistently throughout the text to maintain clarity.

Q14: To promote adoption of RNACOREX, consider uploading the package to the PyPI repository, allowing users to install it via pip. This would eliminate the need for manual environment configuration or file downloads.

A14: To promote adoption of RNACOREX, we've now published the package on PyPI (`https://pypi.org/project/RNACOREX/`), making installation as simple as: `pip install rnacorex`. However, due to file size limitations imposed by PyPI, we were unable to include the large interaction databases that RNACOREX uses for filtering. These databases include TargetScan, DIANA microT, miRTarBase, TarBase and Gencode. They are essential for the package to function correctly and are automatically loaded when the package starts, but must be added after installation.
To address this, we have included a built-in function `download()` that has to be implemented after installing the library. This function will automatically download and save the required databases. To assist users, we also provide another built-in function within the package: `check_engines()`. This function verifies whether the required databases have been correctly added. Once the files are in place, the package will run as intended.

Reviewer 3

Q: Authors propose a Python package that finds disease associated post-transcriptional networks and use them to classify micro and mRNA interactions.

A: We thank the anonymous reviewer for the thoughtful comments. We have carefully addressed all the questions and suggestions raised to the best of our ability and have made the corresponding changes in the manuscript where appropriate.

Q1: My main concern is that the authors propose a new method based on some heuristics but the method is not benchmarked against any existing methods. The paper is positioned as a software paper, a Python package certain features, but the underlying method's validity is not well established. It is not clear if as a user we are using the state of the art method with nice software features.

A1: Thank you for this important observation. The main novelty of our approach lies in the design of the complete pipeline rather than in any single heuristic step, which makes direct benchmarking against existing methods challenging. Broadly speaking, the package consists of two parts: (i) the discovery of co-regulation networks, and, (ii) the construction of a predictive model. Ideally, benchmarking would involve methods that perform both tasks in an integrated manner, but to the best of our knowledge, such methods are scarce. We considered CGBayesNets as a potential reference, since it builds networks from expression data and constructs a Conditional Linear Gaussian model; however, it does not incorporate curated prior knowledge as RNACOREX does. Other tools either focus only on network discovery without prediction, or on predictive modeling without integrated network discovery, making a fair, direct comparison difficult. Therefore, we position RNACOREX as a novel pipeline offering an integrated, curated approach, rather than as an incremental improvement over existing methods.

To clarify this point to the reader, we have added a brief paragraph at the end of the Introduction highlighting these limitations in existing methods and explaining why direct benchmarking with RNACOREX is not straightforward.

Q2: Authors claim the following but there is no experimental support on this: "However, not all miRNAs interact with all mRNAs, and relying solely on expression data often results in a high number of false positives. RNACOREX uses a hybrid approach... This strategy improves accuracy and reduces the likelihood of misleading results..." Please provide evidence that the first approach has many false positives and this new strategy improves accuracy.

A2: We thank the reviewer for this valuable comment. It is well established in the literature that the identification of false positives is a recurrent and significant challenge when working with miRNA–mRNA interaction predictions, especially considering that there are millions of possible miRNA–mRNA interactions. RNACOREX integrates a preprocessing step that significantly narrows down the search space by focusing only on interactions that have been previously predicted or experimentally validated. These candidate interactions are sourced from multiple well-established databases, each of which uses distinct criteria (*e.g.*, seed pairing, structural accessibility, conservation, etc.) for prediction or validation. Therefore, all interactions considered by RNACOREX are supported by evidence. This guarantees that any interaction included in the analysis has a structural or functional basis. Furthermore, the probability of missing a true biologically relevant interaction not captured by any of these prediction engines is extremely low, given the comprehensiveness of the sources used.

In this context, the strategy employed by RNACOREX is both robust and efficient. By restricting the analysis to a biologically grounded universe of candidate interactions, it avoids testing implausible combinations and reduces computational cost. While this may not constitute experimental validation in itself, it represents a biologically informed and methodologically robust way to increase accuracy and reduce misleading results.

Q3: The abstract is confusing with a poor flow. It needs to be restructured. For instance, not clear at all

what this means:

- *"The process is developed using a hybrid approach by combining expert information with empirical knowledge extracted from expression data".*
- *"RNACOREX is a new, easy-to-use tool that allows researchers to find disease associated post-transcriptional networks and use them to classify new unseen observations of micro and mRNA quantifications." Not clear what is classified yet. This is later clarified in classifying tumors.*

Also micro → micro RNA.

We much appreciate the reviewer's feedback. We have revised the abstract to clarify these aspects and to improve its overall flow. By "hybrid method" we refer to the integration of information from different sources to make more robust predictions. As mentioned in previous answer A3, it refers to the combination of curated structural molecular data with empirical analysis of expression data using conditional mutual information. Regarding the second point, RNACOREX is designed to classify any binary outcome variable. In the current given example, it is used to classify patients based on survival time across different cancers. The 'micro' terms have been adapted to micro-RNA (or miRNA).

Q4: Section 2.1 aims to provide an overview but it rather is confusing. Figure 1 and its caption is also confusing. It is not clear what structural information refers to and what is expert information-based score.

A4: Thank you very much for your comment. We agree that the explanation was not sufficiently clear and we have revised the text accordingly.

In our framework, the structural information of an interaction refers to the robustness of prior biological knowledge supporting that interaction, whether it has been computationally predicted or experimentally validated. This is quantified using the structural information score, which reflects both the number of sources (prediction engines or databases) supporting the interaction and the reliability of those sources. This information is considered expert knowledge because it comes from widely-used curated databases such as TargetScan, DIANA, miRTarBase and TarBase. However, we refer to it as structural because the predictions included in these databases are typically based on biological features, especially sequence complementarity between miRNAs and mRNAs, as well as additional molecular criteria such as evolutionary conservation and binding energy. Therefore, in the context of our methodology, expert knowledge and structural information refer to the same concept: a measure of prior evidence based on biologically meaningful, structure-related features and reliable external sources. We have clarified this terminology in the revised manuscript.

Regarding Figure 1, we agree that it was not sufficiently clear and could be confusing, especially for readers unfamiliar with the methodology. To address this, we have revised the figure by simplifying its content and focusing on the key steps of the pipeline. The updated version is designed to improve clarity and better support understanding of the overall workflow.

Q5: Section 2.2 explains structural information score but I do not understand what this needs to do with structure it seems to be a support based on number of databases the interaction occurs and the confidences of those supports.

A5: Thank you for your question. As commented in the previous answer A4, the term "structural" is the one we use to describe information extracted from curated interaction databases. We chose this term because these databases typically rely on sequence-based criteria, meaning that interactions are identified based on the molecular structure of RNA molecules. As you correctly point out, this structural score takes into account two aspects: the number of databases in which a given interaction appears, and the consistency between the pairs of databases reporting that interaction. In this way, the reliability of an interaction is weighted according to how likely each structural evidence is. An interaction found in more databases indicates higher confidence based on its structural basis. Moreover, if these databases are consistent with each

other, the resulting score provides additional robustness, reflecting that the interaction occurs consistently across similar contexts. In contrast, the "functional" score does not rely on molecular structural features, but rather on expression analysis, reflecting the behavior of these molecules during the transcription process.

Q6: Authors divide patients into two classes depending on their survival time (long-survival vs. short-survival) but ensure that there are equal number of samples in each class. Depending on the underlying distribution this may make the time periods meaningless if there is an imbalance in the data. Is this the case?

A6: Thank you for this important observation. As you correctly noted, the cutoff value is not determined by the underlying distribution of survival times but is instead based on a fixed strategy: patients are divided according to the 75th percentile of survival time, with those below this threshold assigned to the "short survival" group and those above (including selected censored cases) to the "long survival" group. This ensures that the cutoff value is adapted to each cancer type while applying the same criterion across datasets, regardless of the specific distribution shape. This approach allows us to maximize the use of available data, particularly by incorporating censored samples when appropriate to balance class sizes. However, we acknowledge that this method does not explicitly model or account for the full underlying survival distribution. It is important to note that this preprocessing step was performed externally and is not part of the core implementation of the RNACOREX package.

Q7: Not clear what this means: "The first two databases include only predicted interactions based on their genomics".

A7: While miRTarBase stores interactions that have been experimentally validated, TargetScan and DIANA-microT store predicted interactions. These predictions are made based on biophysical criteria such as sequence complementarity and evolutionary conservation. We agree that the phrase "based on their genomics" is unclear and does not clearly express what we meant. We have revised the manuscript and removed this sentence.

Q8: L82 first → First.

A8: Thank you for pointing out the typographical error in line 82, where "first" should have been capitalized. We have revised that section of the manuscript, and the text has since been modified, so the error is no longer present. We appreciate your careful reading and helpful feedback.
