# Supplementary material for: RNACOREX - RNA coregulatory network explorer and classifier
Source: PLoS Comput Biol. 2025 Nov 3;21(11):e1013660. doi: 10.1371/journal.pcbi.1013660 (PMC12594346; doi:10.1371/journal.pcbi.1013660)
Supplement: Attachment — Submitted filename: Reviews.pdf [file pcbi.1013660.s008.pdf]

Aitor Oviedo-Madrid
Institute of Data Science and Artificial Intelligence (DATAI), Universidad de Navarra
Ismael Sánchez Bella Building, Campus Universitario
31009 Pamplona, Navarra
Spain

Mark Alber, Ph.D.
Section Editor

PLOS Computational Biology

Dear Editor,

We, the authors, are pleased to present you our reviewed manuscript entitled

RNACOREX - RNA Coregulatory Network Explorer and Classifier

with manuscript number: **PCOMPBIOL-D-24-01999R2**.

We thank you for considering our manuscript and the careful handling of the review process. We provide a point-by-point responses to the Guest Editor and the three anonymous Reviewers.

We confirm that this manuscript is not under consideration elsewhere. The correspondence address is aoviedomadr@unav.es.

Yours sincerely,
Aitor Oviedo-Madrid, José González-Gomariz and Rubén Armañanzas.

RNACOREX - RNA Coregulatory Network Explorer and Classifier

Aitor Oviedo-Madrid, José González-Gomariz and Rubén Armañanzas

Point-by-point response to review comments

Dear Editor,

Here we present our responses to the anonymous reviewers' comments.

- Each question has been addressed as an independent point and appropriately numbered for reference.
- We also attach an amended manuscript with tracked changes in which deleted parts are crossed in ~~strikethrough~~ format, and additions are highlighted in blue color.
- The questions from the Reviewers are typed in *italic* format, whereas our responses are in plain format and **bolded** when a text cites manuscript excerpts.

We would like to thank the anonymous Reviewers for the time and effort in reviewing our work. We have incorporated all suggestions to the best of our ability, increasing the quality and readability of the manuscript. We consider that all concerns were solved in the amended version of the manuscript.

Additional comments

We would like to note that in this revised version we have slightly modified the data preprocessing step by including a log-normalization procedure that was not applied previously. This is a fundamental step in this type of expression analysis, broadly used to stabilize variance and reduce the dominance of highly expressed genes, ensuring a more balanced and reliable identification of miRNA-mRNA interactions. Although this new setup affects some specific numerical results, the overall behavior and biological interpretation remain consistent with the original version. This change does not affect the RNACOREX methodology or implementation, rather it refines the preprocessing pipeline of the analyzed datasets. Additionaly, the PyPI package and the Zenodo repository have been updated to incorporate the changes and improvements made as a result of the reviewers' comments. The code is included in the Github repository at `https://github.com/digital-medicine-research-group-UNAV/RNACOREX`.

Reviewer 1

Q: I want to thank the authors for taking the time to satisfactorily address the comments I raised. After reading the software submission requirements, I understand that an exhaustive biological analysis is not necessary. Even so, I am glad that the other reviewers also requested it. We are not asking for a complex experiment to prove that the predicted interactions are physically occurring, but rather for some interpretability in the model — for example, assessing whether the predicted interactions are related to the phenotypes under study. In the same way the DEG pipeline is proposed, it would be useful to verify that the model is capturing relevant biological information rather than purely topological patterns.

A: We would like to thank the reviewer for their feedback. We fully understand and appreciate the concern regarding the interpretability of the model and the relevance of the predicted interactions to the biological context under study. While the main objective of this work is not to provide an exhaustive biological analysis of each network, RNACOREX allows quantifying how strongly two elements are associated with each other and with the class through the functional information score. This value can be easily accessed from the interaction ranking once the model is trained. To demonstrate that the identified interactions are meaningful, we included two sections on biological discussion. The first one, named "TCGA Pan-Cancer Analysis", included in the main manuscript and in the Supplementary Material, focuses on the most prominent interactions and elements and their known associations with different pathologies. The second one, named "miR-1293 in Head and Neck Squamous Carcinoma", is in the Supplementary Material and provides a more detailed analysis of a subset of relevant interactions, examining their expression patterns and associations with the problem's class. We also include this analysis below (note that Table and Figure numbers differ from those in the supplementary material). These sections demonstrate how RNACOREX results can be used to guide a complex biological investigation.

miR-1293 in Head and Neck Squamous Carcinoma

As an illustrative example, a brief analysis was conducted on some of the most relevant interactions. Supp. Fig. 7 (see supplementary material) shows the most recurrent components across the different networks obtained. Among the tissue-specific miRNAs, miR-1293 stands out, appearing more than 50 times in the HNSC network and representing approximately 50% of that network. Table 1 presents the top 12 interactions in the HNSC interaction ranking. As can be seen, half of these interactions are associated with this miRNA, particularly those included in the ranking based on functional information (calculated from the expression profiles of the elements). Although only the top 12 interactions are shown in the table, expanding the ranking to the entire network reveals that almost all interactions included through functional information are associated with miR-1293. This observation suggests that the expression of this miRNA and its extensive interactions with multiple genes may have a significant impact on HNSC patients.

Supp. Tab 1: Interaction Ranking.

miRNA	mRNA	SS	FS	cor	p_cor	log2FC	p_adj
hsa-mir-451a	DCBLD2	1.000	0.031	0.035	0.530^{ns}	0.416	0.007^{**}
hsa-mir-1293	RNF150	0.204	0.225	-0.554	0.000^{****}	-0.932	$2.48e-4^{***}$
hsa-mir-7706	CCND1	1.000	0.0389	-0.055	0.316^{ns}	0.445	0.024^{*}
hsa-mir-1293	MCF2L	0.204	0.217	-0.551	0.000^{****}	-0.444	0.025^{*}
hsa-mir-3615	ZC3H4	1.000	0.030	-0.035	0.531^{ns}	-0.140	0.019^{*}
hsa-mir-1293	RHBDL3	0.204	0.193	-0.522	0.000^{****}	-0.970	$2.57e-4^{***}$
hsa-mir-4326	OSBPL11	1.000	0.055	-0.150	0.006^{**}	-0.199	0.009^{**}
hsa-mir-1293	CBX7	0.522	0.190	-0.509	0.000^{****}	-0.425	0.004^{**}
hsa-mir-4326	ARRDC3	1.000	0.039	-0.083	0.134^{ns}	-0.293	0.018^{*}
hsa-mir-1293	ABCA3	0.535	0.187	-0.525	0.000^{****}	-0.936	0.001^{**}
hsa-mir-4326	SLC12A6	1.000	0.049	-0.116	0.035^{*}	-0.296	0.034^{*}
hsa-mir-1293	ZNF775	0.204	0.184	-0.522	0.000^{****}	-0.244	0.048^{*}

Table notes. Main 12 interactions in the interaction ranking for the HNSC network. **miRNA:** Interacting miRNA. **mRNA:** Target mRNA. **SS:** Structural score. **FMI:** Functional score. **cor:** Pearson correlation. **p_cor:** p-value of the correlation. **log2FC:** Log fold change of the classes as calculated by PyDeSeq2. **p_adj:** Adjusted p-value of the log fold change after FDR correction. *Asterisks indicate statistical significance: (ns) no significative, (*) $p < 0.05$, (**) $p < 0.01$, (***) $p < 0.001$, (****) $p < 0.0001$.

In Figure 1, the first six interactions related with miR-1293 are presented. The scatter-plot shows the relation between gene and micro expression, while boxplot shows expression differences between classes. As can be seen in the metrics of the graphics, both the correlation and the class differences are always significative. Additionally, there is a negative correlation in all the cases, showing that when miR-1293 has higher expression, the gene tends to underexpress, aligning with the expected biological behaviour of miRNAs, inhibiting the expression of their target genes.

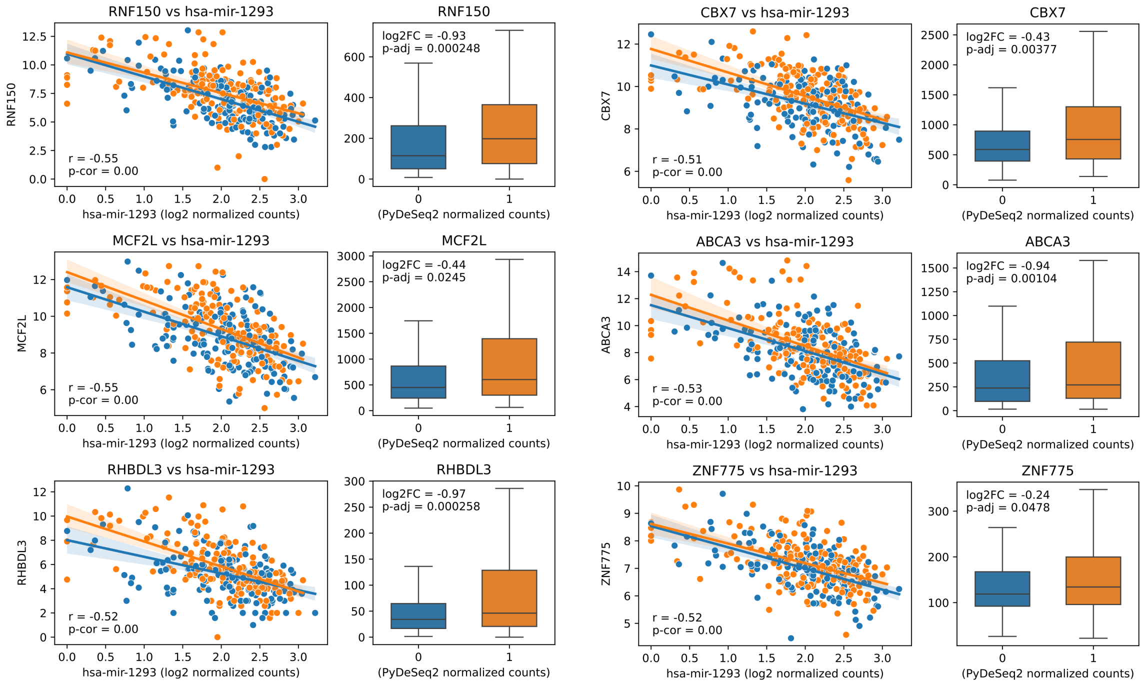

Figure 1: miR-1293 interactions in HNSC. The figure shows the main 6 interactions associated with hsa-mir-1293 in HNSC tissue. For each interaction, a scatter plot displays the relationship between the microRNA and gene expression values, and a boxplot shows their distribution by class. The scatter plots use log-normalized counts, while the boxplots show counts normalized as performed by PyDESeq2 during the differential expression analysis.

To gain deeper insight into each interaction, we examined the behavior of the target genes under conditions of low and high expression of the miR-1293. To this end, miRNA expression was dichotomized into two groups of high and low expression using the expression median. As shown in Figure 2, gene expression patterns differ markedly between these two conditions, especially in miRNA low expression scenarios. In Table 2, some statistics can be accessed. In all cases, the expression of the target gene was higher in the low miRNA expression group, consistent with the negative correlations previously observed. More specifically, when miR-1293 expression was low, the mean expression of the target genes tended to be higher in the long-survival group (Class 1) compared with the short-survival group (Class 0). This difference was statistically significant for MCF2L, RHBDL3, and CBX7, and narrowly missed significance for the remaining three genes. In contrast, under high miRNA expression, differences between survival classes were minimal, except for RNF150, where a small but significant effect persisted.

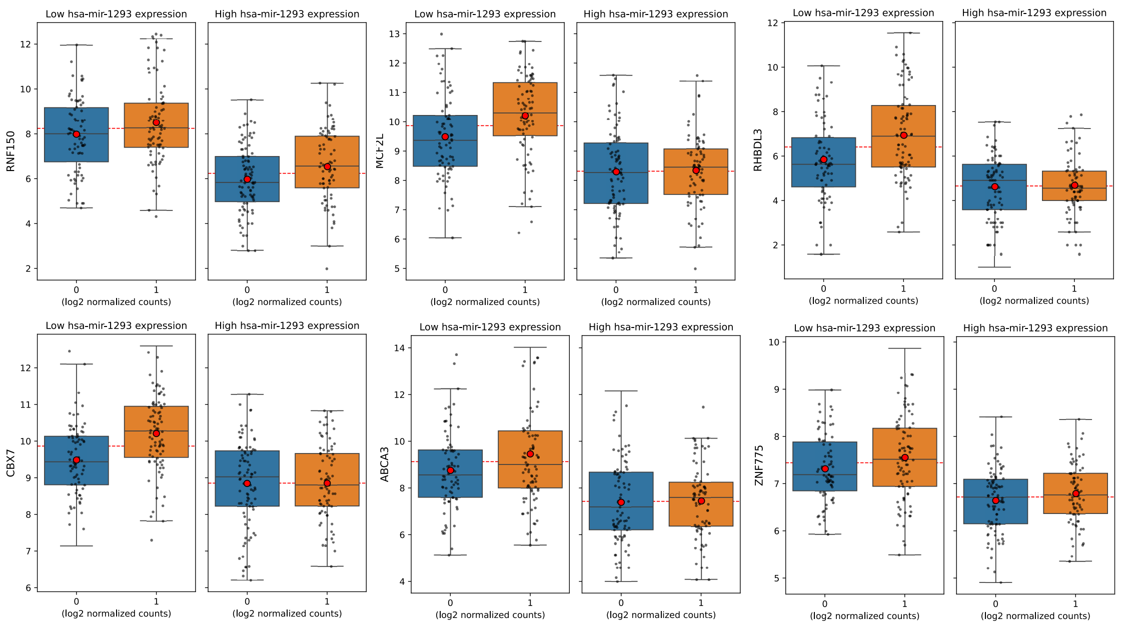

Figure 2: Gene expression levels. The figure shows the expression of six genes associated with hsa-miR-1293, stratified by miRNA expression. Samples are divided into low (below median, left panel) and high (above median, right panel) miRNA expression groups. Within each panel, short-survival samples are shown in blue, and long-survival samples in orange. The red dot indicates the mean expression for each class, and the dashed red line indicates the overall mean expression across both classes within the low or high miRNA group.

Supp. Tab 2: miR-1293 interacting gene metrics.

	Low miR-1293				High miR-1293				
**Gene**	μ_0	μ_1	μ_c	p_c	μ_0	μ_1	μ_c	p_c	p_T
RNF150	7.97	8.49	8.24	0.10^{ns}	5.97	6.53	6.24	0.02^{*}	$4.63e-16^{****}$
MCF2L	9.49	10.21	9.87	$2.77e-4^{***}$	8.29	8.34	8.31	0.60^{ns}	$1.02e-14^{****}$
RHBDL3	5.84	6.92	6.41	$7.25e-4^{***}$	4.63	4.69	4.66	0.99^{ns}	$4.32e-14^{****}$
CBX7	9.49	10.20	9.86	$1.20e-5^{****}$	8.85	8.86	8.85	0.85^{ns}	$4.43e-11^{****}$
ABCA3	8.76	9.46	9.12	0.09^{ns}	7.40	7.46	7.43	0.67^{ns}	$2.87e-12^{****}$
ZNF775	7.32	7.56	7.44	0.07^{ns}	6.65	6.79	6.72	0.21^{ns}	$4.10e-12^{****}$

Table notes. Expression metrics for low and high expression of hsa-mir-1293 (with log2 normalized expression). μ_0: Average expression of class 0 (short-survival) in low / high miRNA expression, μ_1: Average expression of class 1 (long-survival) in low / high miRNA expression, μ_c: Average expression (both classes) for low / high miRNA expression, p_c: p-value of gene expression differences in low- and short-survival for low / high miRNA expression, p_T: p-value of gene expression differences (all data) for low / high miRNA expression. *Asterisks indicate statistical significance: (ns) no significative, (*) $p < 0.05$, (**) $p < 0.01$, (***) $p < 0.001$, (****) $p < 0.0001$.

These results support the hypothesis that miR-1293 plays a relevant regulatory role in HNSC. Moreover, the fact that higher gene expression, particularly under low miRNA expression, is associated with longer survival suggests a potential protective effect of these genes. However, some patients with good survival also show high miR-1293 expression and low gene expression, indicating that survival is not determined solely by these interactions but rather by a more complex set of biological and clinical factors.

Reviewer 3

Q: I thank the authors for addressing my comments. However, some of the issues I raised still remain.

A: We sincerely thank the reviewer for the additional comments and careful reading of our revised manuscript. We appreciate the continued feedback and have addressed the remaining issues in detail to further improve the clarity and quality of the paper.

Q1: Regarding my question on benchmarking the pipeline against others (Q1), the authors reckon that often a method does either network construction or classification. While it is still possible for authors to compare with such methods, they combine multiple tasks and it is understandable to just discuss this issue in the paper.

A1: We appreciate the reviewer's comments acknowledging the challenges of benchmarking RNACOREX. In the revised introduction we have clarified the two main existing approaches: (i) constructing interaction sets using curated biological information; and, (ii) using general-purpose Bayesian network packages for network learning and inference. Comparing RNACOREX with the first type of approaches is problematic: one can study the overlap between interaction sets, but it is difficult to determine quantitatively which set is "better" since such sets cannot be validated outside the laboratory. By contrast, we can compare RNACOREX with generic Bayesian network packages, but this is not entirely fair since these tools use all available data equally without considering the biological relevance of the interactions. Nevertheless, as mentioned in our response to the following question Q2, we have performed a comparative analysis between RNACOREX and CGBayesNets to illustrate the limitations of such generic packages for our specific aim.

Q2: Authors indicate that there is only one method which combines network inference and classification: CGBayesNets. It is different than RNACOREX because it does not include curated. Authors indicate that they are not comparable. I do not understand why this is a problem. CGBayesNets aims to solve the same problem with less data and even uses CLG. So this comparison should show the importance of the prior information and the contribution of the authors to justify the need for a new pipeline.

A2: We thank the reviewer for raising this important point. CGBayesNets is not the only method that combines network inference and classification, as many general-purpose Bayesian network package can develop this functionality over an input dataset. CGBayesNets is specially interesting in our case because it implements Conditional Linear Gaussian (CLG) models, which is exactly the same model implemented in RNACOREX. By using raw data, the package can be employed both to infer a network and to perform classification on new samples. However, it follows a purely data-driven approach, building networks solely from expression data without incorporating curated biological or structural information. In contrast, RNACOREX is specifically adapted to the field of transcriptomics, explicitly integrating biologically and structurally informed knowledge to address domain-specific challenges. As a result, applying CGBayesNets directly to transcriptomic data may lead to limitations that RNACOREX overcomes, highlighting the importance of incorporating prior biological knowledge to generate robust results. To illustrate these limitations, a comparative analysis between RNACOREX and CGBayesNets has been added to *Section 7: Benchmarking* in the Supplementary Material. The analysis is also included below.

Benchmarking

Identifying disease-associated miRNA-mRNA interaction sets is usually approached in one of two ways: (i) using curated biological information, or (ii) applying general-purpose Bayesian network packages for network learning and inference. Comparing RNACOREX with the first type of approach is challenging, since methods based on curated information typically do not have the ability to make predictions on new datasets, and therefore the interactions cannot be validated outside the laboratory. Comparisons with generic Bayesian network packages are also limited, because these tools use all available data without considering the biological relevance of the interactions. Nevertheless, we conducted a comparison with one representative package. CGBayesNets is a Bayesian network software that implements Conditional Linear Gaussian classifiers (CLGs), the same model used in RNACOREX. Unlike RNACOREX, it does not incorporate curated information in

the network learning process. However, it can still infer a network from raw data and make predictions on new datasets. Here, we present a small comparative analysis of RNACOREX and CGBayesNets, highlighting the strengths and limitations of both tools for post-transcriptional co-regulation network analysis.

CGBayesNets. CGBayesNets is a general-purpose package for learning and applying Conditional Linear Gaussian Bayesian Networks from input data, including several structure-learning and inference algorithms. It implements four structure-learning algorithms: K2, Pheno-Centric, Full-Exhaustive, and Naïve Bayes and two inference algorithms: Cowell and Variable Elimination. The package includes a basic pipeline, HybridBN, which uses K2 for structure learning and Cowell for inference. Users can modify parameters, select different algorithms, extract the network, and make predictions as needed. The package can be applied to genomics, metabolomics, and proteomics, learning a network from raw input data and performing inference on new data. However, the implemented algorithms are standard Bayesian network learning and inference methods and are not biologically informed.

Analysis. Unlike CGBayesNets, RNACOREX incorporates curated information on the structure and characteristics of the interactions. To illustrate the importance of this prior information, we conducted a controlled experiment comparing RNACOREX and CGBayesNets. From each of the 13 TCGA databases used in the main experiments of the paper, 50 samples were randomly selected for training and 20 for testing, stratified by class. Both methods were applied using their default pipelines, and evaluated on both the training and independent test sets. Specifically in CGBayesNets, the `HybridBN` script was used for training the networks. The identified network was used for predicting on the test set using `PredictPheno`. In RNACOREX, networks from 2 to 200 interactions were compared using default configuration. The best performing network in the train set (both in accuracy and AUC) was selected and used for predicting over the test set. Results are presented in Table 3. Both CGBayesNets and RNACOREX showed excellent performance on the training data, reaching similar mean accuracies of approximately 93% and AUC-s of 0.98. However, their behavior differed notably when evaluated on the test sets. The accuracy and AUC obtained with RNACOREX were consistently higher than those achieved with CGBayesNets. Across the 13 analyzed datasets, RNACOREX achieved an average accuracy of 61% and AUC of 0.69, whereas CGBayesNets performed only slightly better than random assignment, with an average accuracy of 52% and AUC of 0.51.

Supp. Tab 3: Benchmarking.

	CGBayesNets			RNACOREX		
**Disease**	Acc	AUC	k	Acc	AUC	k
BRCA	0.94 / 0.65	1.00 / 0.61	24	0.98 / 0.60	0.99 / 0.70	133 / 131
COAD	0.88 / 0.55	0.93 / 0.54	16	0.92 / 0.90	0.99 / 0.91	94 / 175
HNSC	0.90 / 0.55	0.97 / 0.50	17	0.82 / 0.35	0.95 / 0.55	7 / 176
KIRC	0.96 / 0.55	1.00 / 0.49	22	0.90 / 0.75	0.96 / 0.81	48 / 69
LAML	0.96 / 0.50	1.00 / 0.56	37	0.96 / 0.80	0.99 / 0.89	147 / 147
LGG	0.94 / 0.60	1.00 / 0.60	32	0.90 / 0.50	0.97 / 0.70	190 / 143
LIHC	0.96 / 0.45	1.00 / 0.40	32	0.96 / 0.65	0.98 / 0.71	124 / 126
LUAD	0.90 / 0.40	0.95 / 0.35	14	0.98 / 0.55	1.00 / 0.68	61 / 60
LUSC	0.92 / 0.40	0.96 / 0.37	19	0.96 / 0.65	0.99 / 0.65	193 / 199
SARC	0.84 / 0.50	1.00 / 0.54	11	0.96 / 0.70	0.99 / 0.77	72 / 192
SKCM	0.96 / 0.50	0.96 / 0.66	29	0.94 / 0.50	0.99 / 0.55	70 / 98
STAD	0.98 / 0.60	1.00 / 0.51	35	0.96 / 0.40	0.99 / 0.47	140 / 167
UCEC	0.94 / 0.50	0.98 / 0.49	16	0.92 / 0.55	0.97 / 0.61	82 / 70

Table notes. Classification metrics for CGBayesNets and RNACOREX in the 13 databases. Accuracy and AUC are shown in (train / test) format. k represents the number of interactions of the selected network. In RNACOREX, k is defined as (best accuracy network / best AUC network) format.

To assess whether these performance differences were statistically significant, we conducted a significance analysis comparing the test set results from both methods. The Shapiro–Wilk test did not reject normality

for any of the outputs, although the small number of observations (n = 13) limits the strength of this conclusion. For accuracy, the evidence was insufficient to confirm a consistent difference between methods, as p-values were slightly above 0.1 (p = 0.1104 for the paired t-test and p = 0.1254 for the Wilcoxon test). In contrast, the AUC differences were statistically significant, indicating that RNACOREX achieved superior performance on the test data (p = 0.0012 for the paired t-test and p = 0.0034 for the Wilcoxon test).
These results highlight the advantages of incorporating biological constraints into network inference. CGBayesNets relies solely on statistical associations, including all interactions that appear relevant, regardless of biological plausibility. Without biological constraints, unrealistic interactions can appear, such as mRNA–miRNA links, if their expression patterns seem relevant. This makes the networks fit the input data very well but often leads to overfitting and poor generalization to new datasets. RNACOREX, by contrast, filters interactions according to biological and structural criteria, reducing the number of input variables and producing networks that are both biologically more meaningful and better able to generalize beyond the training data.

This analysis is also referenced in the results of the main manuscript: **"In addition to the main results of the article, the performance of RNACOREX has also been compared with CGBayesNets, a generic Bayesian network package that also implements CLGs. The results show the advantages of using a pipeline like RNACOREX, specifically adapted to this problem, over a generic Bayesian network model. The results are presented in the *Section 7: Benchmark* in the supplementary material."**

Q3: Regarding my comment (Q2), could the authors cite references from the literature to back up the claim that "It is well established in the literature that the identification of false positives is a recurrent and significant challenge when working with miRNA–mRNA interaction predictions".

A3: We thank the reviewer for the suggestion to reinforce this point with additional references. There is extensive literature documenting the challenge of false positives in computational miRNA–mRNA target prediction in the absence of experimental validation. For example, Pinzón et al. (*"microRNA target prediction programs predict many false positives,"* Genome Research, 2017) reported that many targets identified solely through evolutionary conservation and seed matches are unlikely to represent true biological interactions. Similarly, Fridrich et al. (*"Too many false targets for microRNAs: Challenges and pitfalls in prediction of miRNA targets and their gene ontology in model and non-model organisms,"* Bioessays, 2019) mentioned that the reliability of predicted miRNA–mRNA interactions remains limited without experimental confirmation. In our manuscript we already cite Muniategui et al. (*"Joint Analysis of miRNA and mRNA expression data,"* Briefings in Bioinformatics, 2013), who showed that the false positive rate of leading computational approaches is high, and that integrating expression data can help filter predictions and increase robustness. Likewise, Saito et al. (*"MicroRNAs – targeting and target prediction,"* New Biotechnology, 2010) noted that incorporating expression information provides additional evidence for interaction support and can substantially reduce spurious predictions.

RNACOREX has been developed within this context: rather than relying exclusively on structural features or expression data, it integrates both types of information to mitigate the problem of false positives and to strengthen the reliability of the resulting interaction sets. In the revised version, the Introduction section expands on the discussion about the challenges of making predictions using only expression or structural data, and how combining both approaches can overcome these limitations. The work of Saito et al. is now cited along with Muniategui et al. in order to strengthen the explanation: **"This hybrid approach has demonstrated its ability to filter spurious interactions and identify more robust interaction sets"**. Additionally, the work of Pinzón et al. is now cited pointing out the problem of relying only on structural or expression data: **"However, not all miRNAs interact with all mRNAs, and relying solely on expression or structural data often results in a high number of false positives"**.

Q4: The issue in Section 2.1 remains: It is not clear what "structural information" is at this point in "Candidate interactions are initially filtered based on their structural information". This sounds like the 2D/3D structure of the RNAs but it is not and it is confusing. Please clarify in text with you answer to Q5.

A4: We thank the reviewer for pointing out this source of potential confusion. The structural information score relies on the presence of an interaction in resources such as TargetScan, DIANA, miRTarBase, and TarBase, assigning higher scores to interactions consistently supported across these sources. This presence is considered an indicator of the "structural" nature of the interaction because predictions in TargetScan and DIANA incorporate features related to sequence complementarity, regional accessibility, and target site position (see *Section 2: Engines* in the Supplementary Material). These features are strongly influenced by the RNA secondary and even tertiary structure, although they do not directly represent the complete 2D/3D structure of the molecule. The concepts of structural and functional information are briefly introduced in the manuscript just before *Section 2.1 RNACOREX Pipeline*, in the introduction of *Section 2 Design and Implementation*. The text has been revised to better clarify the nature of each concept, defining functional information as **"empirical expression values"** and structural information as **"curated knowledge about the molecular structure of miRNAs and mRNAs"**. In *Section 2.3 Information Scores*, the concept of structural information is further elaborated, showing how this curated information is quantified based on the structural features of the interaction. At this point, an additional clarification is included: **"The term 'structural' is used because the predictions generated by these tools are based on features such as sequence complementarity, regional accessibility, and target site position. Therefore, if an interaction has been consistently predicted, it is likely due to its structural characteristics"**.

Q5: Abstract: while offers —¿ while offering

A5: Thank you for the correction. It is fixed accordingly.

Reviewer 4

Q: The paper introduces RNACOREX, a graph-based framework that integrates known miRNA–mRNA interaction databases with expression data to build coregulation networks. Using a Conditional Linear Gaussian classifier, RNACOREX classifies patient samples while simultaneously highlighting potentially disease-relevant regulatory interactions. The method allows users to control network construction through different interaction-ranking strategies and number of edges, balancing predictive accuracy with biological interpretability.

A: We would like to sincerely thank the reviewer for the time and effort dedicated to evaluating our manuscript. We greatly appreciate the constructive comments and suggestions, which have helped us to improve the clarity and rigor of the work. We have carefully addressed each of the points raised and made the corresponding changes in the manuscript.

Q1: The performance evaluation of RNACOREX may be confounded by the fact that Differential Expression Analysis (DEA) is performed using the long and short survival group labels. Since DEA already incorporates class information, the subsequent network construction cannot fully demonstrate that the model itself contributes to classification performance. In principle, one could randomly select mRNAs, perform DEA, and then use a standard classifier (e.g., SVM) to obtain comparably good results. The authors should clarify how the results would change if DEA were not performed as a preprocessing step, or provide an analysis demonstrating that the observed performance is not solely driven by the initial DEA filtering.

A1: We thank the reviewer for this insightful comment. To ensure a fair comparison, all models evaluated in the manuscript were trained using exactly the same variables, selected among the differentially expressed genes. Thus, the differences in performances come from the models themselves, not from the previous DEA selection. Moreover, the DEA process is only an example performed for the experiments described in the paper, and not an actual functionality of the package itself. Users are free to select the input data for the package in any way they consider appropriate, without the need to perform a DEA previously. However, running the model without a prior DEA would considerably increase the computational complexity, as it would involve a much larger number of potential interactions that are otherwise filtered out by the DEA step.
From the results, it cannot be said that the Conditional Linear Gaussian implemented by RNACOREX is clearly superior to other vector-based classifiers, as the observed performance is comparable. However, CLG, being a graphical model, offers explainability advantages that other traditional classifiers including SVM, Random Forest, or Gradient Boosting lack. Other graphical models were also trained using the same post-DEA variables, yet they performed noticeably worse than the CLG. This suggests that the observed performance improvement is not due to the DEA-based feature selection alone. Anyway, it is important to emphasize that the main innovation of RNACOREX does not lie alone in the CLG modeling but rather in how the network structure is induced, combining expression data with expert knowledge on micro-gene interactions. This specific pipeline allows the model to be transparent, interpretable, and competitive in predictive metrics, making it well-suited to this specific problem.

Q2: In the introduction, the authors do not clearly articulate the primary objective of RNACOREX. Classifying patients according to survival time should be presented as a validation strategy rather than as the central aim of the model. Based on the structure of the paper, it appears that the ultimate goal is to identify miRNA–mRNA interactions and potential gene groups associated with specific diseases. If this interpretation is correct, the authors should explicitly state this goal in the abstract/introduction to better frame the contribution and novelty of the work.

A2: Thank you very much for your helpful comments. We agree that in the manuscript the classification of new samples is mainly used to check and validate the set of interactions we found. However, we also want to emphasize that the classification of new samples is not only a way to validate our results, but also an important function of the package itself, and one of its main goals. We see two main goals in RNACOREX: (1) finding sets of interactions linked to specific diseases or biological conditions; and, (2) using these sets to

predict results in new samples. We tried to make this dual purpose clear in the abstract and introduction, as we do not reckon one goal is more important than the other.

Q3: The claim of "successfully uncovering disease-specific networks" appears overstated. As reported, the model's performance is only comparable to, or in some cases weaker than, existing machine learning approaches. Moreover, the current implementation of RNACOREX does not yet provide substantive insights into underlying pathology. The authors should temper this statement and clarify that such interpretability remains a potential future direction rather than a current strength of the model.

A3: Thank you very much for your comment. Regarding model performance, our intention was not to claim superiority over existing machine learning approaches, but rather to emphasize that RNACOREX achieves competitive performance, comparable to widely used models, while substantially outperforming other interpretable approaches such as GNNs and Graph Kernels. In line with this, the abstract describes RNACOREX as having **"achieved competitive predictive performance compared to widely used classification algorithms"** and in the results section we further note that it **"achieved similar results to vector-based classifiers."**

Regarding the model's interpretability, the extracted interaction set corresponds to those that RNACOREX identifies as most broadly associated with each phenotype. In Section *3.4. TCGA Pancancer Analysis*, we briefly describe the most recurrent interactions and their previously reported associations with different cancers. This analysis is intentionally general and does not aim to provide an in-depth examination of interaction sets for each tissue individually. We designed the study in this way because the primary goal of the manuscript is to demonstrate the overall usability of the tool; nonetheless, we acknowledge that a detailed, tissue-specific analysis of each network is possible and represents a logical next step for future research. In Supplementary Material Section *6.3. miR-1293 in Head and Neck Squamous Carcinoma* we have included a brief study of some of the most relevant interactions, illustrating how RNACOREX results can be explored in greater biological detail. This study is also attached at the end of this response.

We also recognize that the phrase *"The tool successfully uncovered disease-specific networks"* and the surrounding paragraph may sound overly optimistic and are not fully supported by the limited biological analysis presented. To address the reviewer's concerns, we revised the text to remove potentially overstated claims and to adopt a more precise and balanced formulation: **"We tested RNACOREX on 13 different cancer types using data from The Cancer Genome Atlas. The tool revealed disease-associated networks and achieved performance comparable to other popular machine learning models. While the current implementation does not yet provide definitive biological insights, it highlights molecular interactions most associated with each phenotype, pointing to a promising direction for future investigations connecting prediction with biological context."**

miR-1293 in Head and Neck Squamous Carcinoma

As an illustrative example, a brief analysis was conducted on some of the most relevant interactions. Supp. Fig. 7 (see supplementary material) shows the most recurrent components across the different networks obtained. Among the tissue-specific miRNAs, miR-1293 stands out, appearing more than 50 times in the HNSC network and representing approximately 50% of that network. Table 4 presents the top 12 interactions in the HNSC interaction ranking. As can be seen, half of these interactions are associated with this miRNA, particularly those included in the ranking based on functional information (calculated from the expression profiles of the elements). Although only the top 12 interactions are shown in the table, expanding the ranking to the entire network reveals that almost all interactions included through functional information are associated with miR-1293. This observation suggests that the expression of this miRNA and its extensive interactions with multiple genes may have a significant impact on HNSC patients.

Supp. Tab 4: Interaction Ranking.

miRNA	mRNA	SS	FS	cor	p_cor	log2FC	p_adj
hsa-mir-451a	DCBLD2	1.000	0.031	0.035	0.530^{ns}	0.416	0.007^{**}
hsa-mir-1293	RNF150	0.204	0.225	-0.554	0.000^{****}	-0.932	$2.48e-4^{***}$
hsa-mir-7706	CCND1	1.000	0.0389	-0.055	0.316^{ns}	0.445	0.024^{*}
hsa-mir-1293	MCF2L	0.204	0.217	-0.551	0.000^{****}	-0.444	0.025^{*}
hsa-mir-3615	ZC3H4	1.000	0.030	-0.035	0.531^{ns}	-0.140	0.019^{*}
hsa-mir-1293	RHBDL3	0.204	0.193	-0.522	0.000^{****}	-0.970	$2.57e-4^{***}$
hsa-mir-4326	OSBPL11	1.000	0.055	-0.150	0.006^{**}	-0.199	0.009^{**}
hsa-mir-1293	CBX7	0.522	0.190	-0.509	0.000^{****}	-0.425	0.004^{**}
hsa-mir-4326	ARRDC3	1.000	0.039	-0.083	0.134^{ns}	-0.293	0.018^{*}
hsa-mir-1293	ABCA3	0.535	0.187	-0.525	0.000^{****}	-0.936	0.001^{**}
hsa-mir-4326	SLC12A6	1.000	0.049	-0.116	0.035^{*}	-0.296	0.034^{*}
hsa-mir-1293	ZNF775	0.204	0.184	-0.522	0.000^{****}	-0.244	0.048^{*}

Table notes. Main 12 interactions in the interaction ranking for the HNSC network. **miRNA:** Interacting miRNA. **mRNA:** Target mRNA. **SS:** Structural score. **FMI:** Functional score. **cor:** Pearson correlation. **p_cor:** p-value of the correlation. **log2FC:** Log fold change of the classes as calculated by PyDeSeq2. **p_adj:** Adjusted p-value of the log fold change after FDR correction. *Asterisks indicate statistical significance: (ns) no significative, (*) $p < 0.05$, (**) $p < 0.01$, (***) $p < 0.001$, (****) $p < 0.0001$.

In Figure 3, the first six interactions related with miR-1293 are presented. The scatter-plot shows the relation between gene and micro expression, while boxplot shows expression differences between classes. As can be seen in the metrics of the graphics, both the correlation and the class differences are always significative. Additionally, there is a negative correlation in all the cases, showing that when miR-1293 has higher expression, the gene tends to underexpress, aligning with the expected biological behaviour of miRNAs, inhibiting the expression of their target genes.

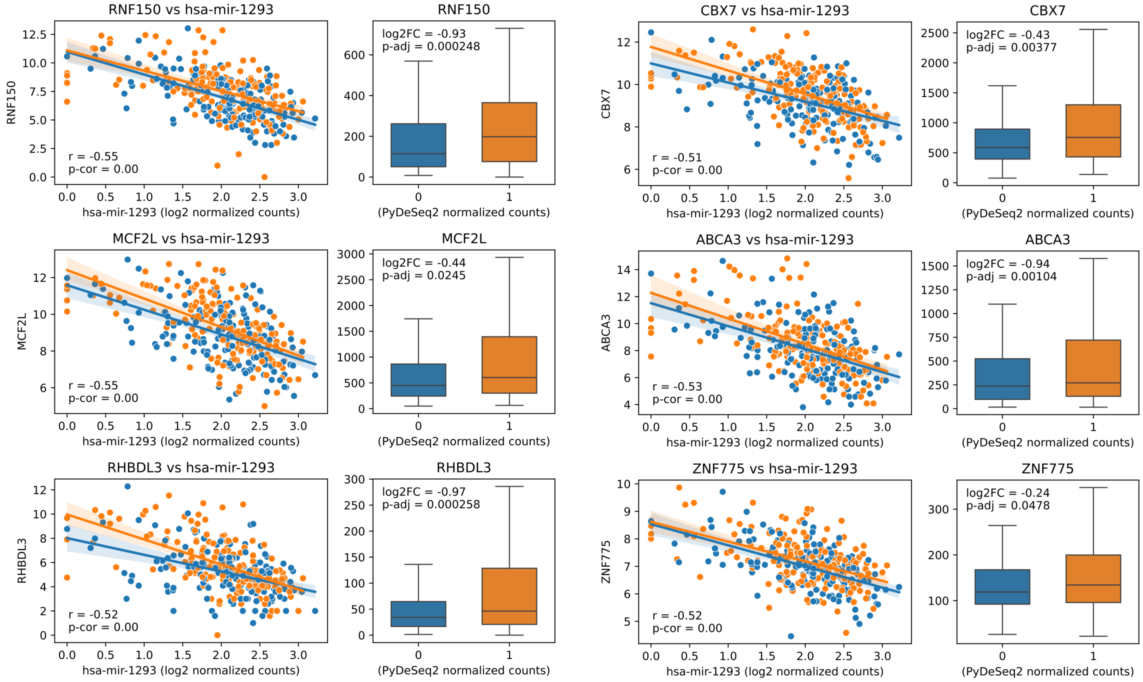

Figure 3: miR-1293 interactions in HNSC. The figure shows the main 6 interactions associated with hsa-mir-1293 in HNSC tissue. For each interaction, a scatter plot displays the relationship between the microRNA and gene expression values, and a boxplot shows their distribution by class. The scatter plots use log-normalized counts, while the boxplots show counts normalized as performed by PyDESeq2 during the differential expression analysis.

To gain deeper insight into each interaction, we examined the behavior of the target genes under conditions of low and high expression of the miR-1293. To this end, miRNA expression was dichotomized into two groups of high and low expression using the expression median. As shown in Figure 4, gene expression patterns differ markedly between these two conditions, especially in miRNA low expression scenarios. In Table 5, some statistics can be accessed. In all cases, the expression of the target gene was higher in the low miRNA expression group, consistent with the negative correlations previously observed. More specifically, when miR-1293 expression was low, the mean expression of the target genes tended to be higher in the long-survival group (Class 1) compared with the short-survival group (Class 0). This difference was statistically significant for MCF2L, RHBDL3, and CBX7, and narrowly missed significance for the remaining three genes. In contrast, under high miRNA expression, differences between survival classes were minimal, except for RNF150, where a small but significant effect persisted.

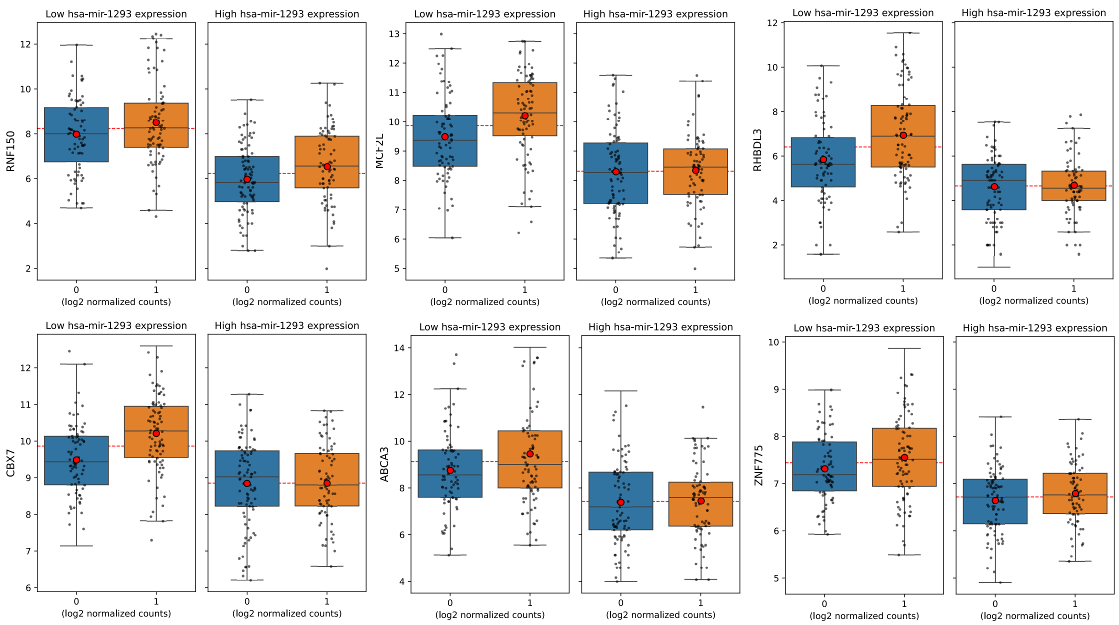

Figure 4: Gene expression levels. The figure shows the expression of six genes associated with hsa-miR-1293, stratified by miRNA expression. Samples are divided into low (below median, left panel) and high (above median, right panel) miRNA expression groups. Within each panel, short-survival samples are shown in blue, and long-survival samples in orange. The red dot indicates the mean expression for each class, and the dashed red line indicates the overall mean expression across both classes within the low or high miRNA group.

Supp. Tab 5: miR-1293 interacting gene metrics.

	Low miR-1293				High miR-1293				
**Gene**	μ_0	μ_1	μ_c	p_c	μ_0	μ_1	μ_c	p_c	p_T
RNF150	7.97	8.49	8.24	0.10^{ns}	5.97	6.53	6.24	0.02^{*}	$4.63e-16^{****}$
MCF2L	9.49	10.21	9.87	$2.77e-4^{***}$	8.29	8.34	8.31	0.60^{ns}	$1.02e-14^{****}$
RHBDL3	5.84	6.92	6.41	$7.25e-4^{***}$	4.63	4.69	4.66	0.99^{ns}	$4.32e-14^{****}$
CBX7	9.49	10.20	9.86	$1.20e-5^{****}$	8.85	8.86	8.85	0.85^{ns}	$4.43e-11^{****}$
ABCA3	8.76	9.46	9.12	0.09^{ns}	7.40	7.46	7.43	0.67^{ns}	$2.87e-12^{****}$
ZNF775	7.32	7.56	7.44	0.07^{ns}	6.65	6.79	6.72	0.21^{ns}	$4.10e-12^{****}$

Table notes. Expression metrics for low and high expression of hsa-mir-1293 (with log2 normalized expression). μ_0: Average expression of class 0 (short-survival) in low / high miRNA expression, μ_1: Average expression of class 1 (long-survival) in low / high miRNA expression, μ_c: Average expression (both classes) for low / high miRNA expression, p_c: p-value of gene expression differences in low- and short-survival for low / high miRNA expression, p_T: p-value of gene expression differences (all data) for low / high miRNA expression. *Asterisks indicate statistical significance: (ns) no significative, (*) $p < 0.05$, (**) $p < 0.01$, (***) $p < 0.001$, (****) $p < 0.0001$.

These results support the hypothesis that miR-1293 plays a relevant regulatory role in HNSC. Moreover, the fact that higher gene expression, particularly under low miRNA expression, is associated with longer survival suggests a potential protective effect of these genes. However, some patients with good survival also show high miR-1293 expression and low gene expression, indicating that survival is not determined solely by these interactions but rather by a more complex set of biological and clinical factors.

Q4: RNACOREX integrates four datasets, but the manuscript does not explain how potential bias or batch effects across these datasets are addressed. Furthermore, it would be informative if the authors could report how many miRNA–mRNA interactions are shared across datasets and how many are unique to each source. This additional analysis would provide users with a clearer understanding of the consistency and complementarity of the datasets employed.

A4: We thank the reviewer for this valuable comment. In RNACOREX, two databases of predicted interactions and two of experimentally validated interactions are considered. Although each employs different methodologies, this diversity is not regarded as a limitation but rather as an added value, since interactions consistently identified across multiple databases can be considered more reliable, having been detected independently by different approaches. In addition, since these resources are based on independent computational predictions rather than comparable experimental measurements, they are not subject to a conventional batch effect that would require correction.
To address the reviewer's suggestion, we have added a new subsection entitled *"Engine Curation"* within *"Section 1: "Engines"* in the Supplementary Material, which now provides a detailed description of the integration process and the composition of each database. In addition, we have included a summary of the overlap between the datasets, reporting how many miRNA–mRNA interactions are shared across databases and how many are unique to each source. This additional analysis should give users a clearer understanding of the consistency and complementarity of the datasets we used.

Q5: In line 142, the authors state that "only those interactions that are supported by at least one of the previously mentioned sources" are retained. However, in the definition of the SI score (line 186), it is stated that "the interaction must be present in at least two databases to be considered." Does this mean that SI scores are only computed for interactions present in at least two sources, while interactions supported by only one source are assigned only a functional information score without any SI score? Clarification is needed.

A5: We sincerely thank the reviewer for this precise and very helpful observation. You are absolutely right: during the initial filtering we only remove interactions that are absent from all engines, but when calculating the structural information score, any interaction present in only a single engine is automatically assigned a score of zero. To clarify this point, we have revised the manuscript to explicitly state: **"Interactions that appear only in a single engine may still remain at the stage of calculating structural information. However, since they are not supported by at least two engines, their structural information is automatically assigned to zero, as they cannot be considered as consistently predicted or validated interactions."** As already noted in the text, the functional information score is then computed only for interactions with a non-zero structural information score: **"For all interactions with a non-zero *structural information score*, the *functional information score* is computed."** This procedure ensures that interactions supported by only one engine are effectively discarded while also reducing computational complexity, since the mutual information computation is avoided for those cases. In practice, the outcome is the same as discarding from the outset all interactions not present in at least two engines.

Q6: In line 217, the authors describe a tie-breaking strategy. Have alternative tie-breaking methods been considered or tested, and if so, how do the results compare?

A6: We thank the reviewer for raising this point. The original tie-breaking method has not been formally compared with alternative approaches. This method prioritizes the most isolated interactions in the event of a tie, aiming to incorporate the most novel information into the network. We recognize that alternative strategies may be of interest in certain cases. To address this, we have included two additional tie-breaking methods in the revised version of the package. The first, referred to as the *'connected'* method, operates in the opposite manner to the original, prioritizing the most connected interactions when ties occur. The second, called the *'functional'* method, resolves ties using functional information to rank interactions. A detailed explanation of all three methods is provided in the *Subsection 3.4. Tie-breaking Procedures* of the Supplementary Material. Users are encouraged to determine which method best suits their data and research objectives. The next figure (also included in supplementary material), shows the differences between three networks built with the same number of interactions (k) and a different tie-breaking methods.

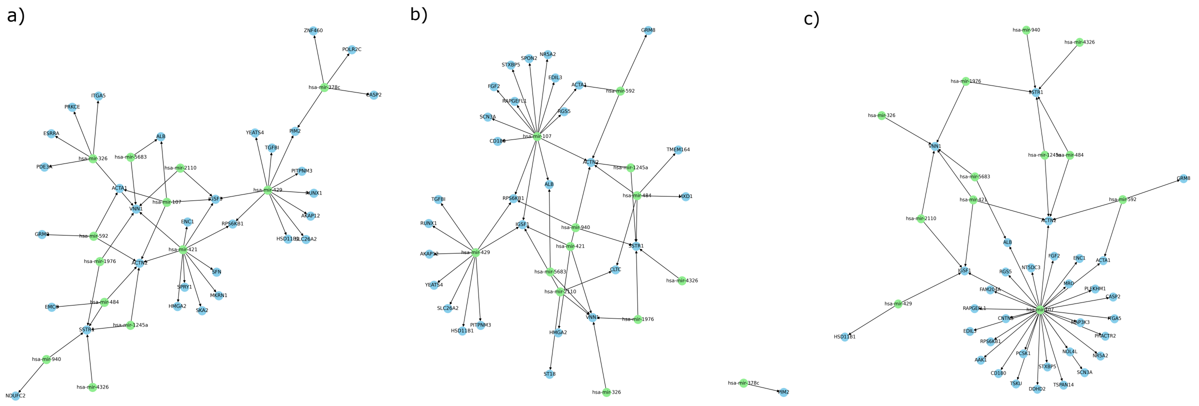

Figure 5: Tie-breaking procedures. Three networks with the same number of interactions are shown, constructed using the same input data (BRCA dataset) and different tie-breaking methods: **a) Isolated method. b) Functional method. c) Connected method.**

Q7: In the Conditional Linear Gaussian Classifier section (line 250), the class variable C is not clearly defined. This should be explicitly described in the main text rather than left only to the supplementary material.

A7: You're right that the definition of the class variable C can sometimes be overlooked. In *Section 2.1 RNACOREX Pipeline*, when defining the input dataset of the model the phrase **"and a binary class or phenotype"** is added. In the definition of the functional information score **"regarding the class C"** is included when defining the conditional mutual information. In the Conditional Linear Gaussian section, the variable C is defined as a **"binary class variable (phenotype)"** and is explicitly stated to be **"treated as a parent of all other variables"**.

Q8: Line 352 mentions that the model was trained using a 3-fold cross-validation. Have the authors considered alternative cross-validation strategies, and what was the rationale for choosing 3-fold in particular?

A8: We appreciate the reviewer's comment. The cross-validation strategy is not the focus of this work, since the main goal of the paper is to present and evaluate the RNACOREX framework itself. For illustrative purposes, we applied a 3-fold cross-validation as a reasonable compromise given the limited sample size of the dataset, in order to balance training data availability with validation reliability. Of course, alternative cross-validation schemes (*e.g.*, 5-fold, 10-fold, or leave-one-out) could also be run and may be explored in future work. However, the choice of cross-validation does not affect the methodology implemented in the package, which remains independent of this evaluation step.
